# New and poorly known Holarctic species of *Boletina* Staeger, 1840 (Diptera, Mycetophilidae)

**DOI:** 10.3897/BDJ.4.e7218

**Published:** 2016-01-18

**Authors:** Jukka Salmela, Anna Suuronen, Kari M Kaunisto

**Affiliations:** ‡ Natural Heritage Services (Metsähallitus), Rovaniemi, Finland; § Zoological Museum, Department of Biology, University of Turku, Finland; | University of Jyväskylä, Dept. of Biological and Environmental Sciences, Jyväskylä, Finland; ¶ Jyväskylä University Museum, University of Jyväskylä, Jyväskylä, Finland; # Zoological Museum, Department of Biology, University of Turku, Turku, Finland

**Keywords:** Boreal zone, arctic zone, fungus gnats, taxonomy, species richness

## Abstract

**Background:**

The genus *Boletina* is a species rich group of fungus gnats. Members of the genus are mainly known from temperate, boreal and arctic biomes. Phylogeny of the genus is still poorly resolved, dozens of species are insufficiently described and undescribed species are often discovered, especially from samples taken from the boreal zone.

**New information:**

Four new species are described. *Boletina valteri* Salmela sp.n. (Finland), *Boletina kullervoi* Salmela sp.n. (Finland), *B. hyperborea* Salmela sp.n. (Finland, Norway, Sweden, Canada) and *B. nuortti* Salmela sp.n. (Finland). *Boletina arctica* Holmgren is redescribed and reported for the first time from the Canadian high arctic zone. *Boletina borealis* Zetterstedt and *B. birulai* Lundström are reported for the first time from Canada. *Boletina subnitidula* Sasakawa (syn. n.) is proposed as a junior synonym of *B. pallidula* Edwards.

## Introduction

**Figure 1.**
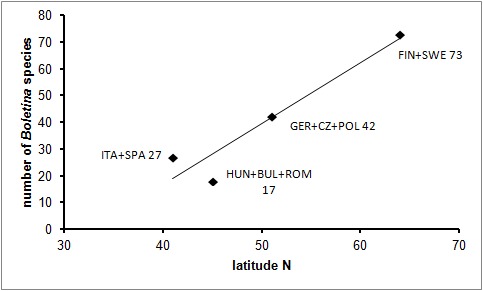
A graph showing the relationship of latitude (x-axis) to the number of *Boletina* species in continental Europe. ITA+SPA=Italy and Spain (27 spp), HUN+BUL+ROM=Hungary, Bulgaria and Romania (17 spp), GER+CZ+POL=Germany, Czech Republic and Poland (42 spp), FIN+SWE=Finland and Sweden (73 spp). Species numbers are based on Fauna Europaae (Chandler 2014) and subsequent additions, such asParvu 2002, Ševčík and Papp 2002, Parvu 2004, Schumann 2004, Kurina 2008, Ševčík and Vonička 2008, Bechev 2010. Fennoscandian (FIN+SWE) number is based on Kjaerandsen 2015, and unpublished records of 15 undescribed species (Kjaerandsen, Polevoi, Salmela & Söli, in prep.). The adjacent European countries were combined in order to reduce variation in their geographical areas. Latitudes represent rough midpoints of the respective groups of countries.

The genus *Boletina* Staeger, 1840 (Staeger 1840) belongs to the family Mycetophilidae and its subfamily Gnoristinae (Väisänen 1986, Rindal et al. 2009, or Gnoristini sensu Edwards 1925, Söli 1997). There are 137 recent *Boletina* species, of which 99 are Palaearctic, 11 Oriental and 34 Nearctic. Only seven species have a Holarctic range and 74 of the Palaearctic species are present in Europe (Suppl. material 1). It should be noted that the numbers presented above are expected to increase, as several Fennoscandian species await description (Fig. 1) and in the Nearctic region the genus is very poorly known (Taber 2011, Taber 2013, J. Salmela pers.obs.). Furthermore, 31 (23 %) out of all described species either are known only from female specimens or their descriptions are too deficient for an understanding of their morphology and thus allowing positive identification of the taxa (Suppl. material 1​). In Europe the genus seems to have an increasing number of species toward higher latitudes, that is, there are more species in Fennoscandia than in either Central Europe or the Mediterranean region (Fig. 1​). The boreal zone may harbor the highest number of *Boletina* species, but several species are also known to occur on the subarctic and Arctic zones (Lundström 1915, Zaitzev 1994, Polevoi 2013). It should be noted, however, that the number of conducted faunistic studies is higher in Fennoscandia than in southern Europe. Furthermore, some *Boletina* species display boreo-mountainous distribution pattern (see e.g. Kurina 2008), being absent from lowland areas; these both factors may obscure the latitudinal pattern presented here.

Species of the genus can be distinguished by the following morphological characteristics: wing membrane without macrotrichia; mediotergite bare; laterotergite haired or bare; Sc ending in C; Sc2 present or absent; R4 absent; Sc ending in C beyond level of base of crossvein ta; mouthparts shorter than height of head (modified from Söli et al. 2000, see also Saigusa 1968). In addition the male hypopygium has a pair of ventral lobes, here termed as sternal submedian appendages of gonocoxites, that have great value in species identification. Gonostyli may consist of one (e.g. all species described here) or two lobes (e.g. *Boletina dubia* (Meigen), *B. landrocki* Edwards, *B. polaris* Lundström). Among species with a single lobe of gonostylus, the gonostylus mostly has a setose and bulbose basal part (here termed as outer branch) and a narrower glabrous branch bearing a few strong apical spines or setae (inner branch). The cercus is conspicuous, bearing characteristic combs (i.e. stout darkened setae arranged in more or less regular rows or scattered on the dorsal surface). The aedaegal complex is more or less firmly attached to the sternal submedian appendage of gonocoxites via lateral branches or ventrodistal sclerites of the aedeagus (Sasakawa and Kimura 1974). Parameres are highly varied and mostly symmetrical (asymmetrical only in *B. cornuta* Zaitzev). However, the genus is paraphyletic (Martinsson et al. 2011), and monophyletic groups within the genus may deserve generic ranks of their own (see e.g. Martinsson and Kjaerandsen 2012).

Immature stages of the genus are extremely poorly known. In fact, notes on larval stages or rearing records are available for only nine species (Suppl. material 2), all of which are European species. Based on these records, the species are associated with decaying wood, soil, leaf litter and liverworts; interestingly only one species has been once reared from fungi (*Suillus bovinus*, Boletales) (Suppl. material 2). However, some of these records may represent pupation places, not real larval microhabitats (​Martinsson et al. 2011). In the boreal zone, *Boletina* may be one of the most abundant groups of fungus gnats in entomological samples collected from forests (J. Salmela, pers.obs.). However, *Boletina* may also be abundant in treeless habitats such as mountains and mires (Edwards 1925, Jakovlev et al. 2014).

In the present paper we describe four new *Boletina* species (three from Finland, one from Fennoscandia and Canada). In addition, one new synonymous name is proposed and one species is reported for the first time from the Nearctic region.

## Materials and methods

All studied Fennoscandian specimens were obtained from Malaise trap samples and are stored in 70 % ethanol. Material deposited in CNC is dry and pinned. The morphological terminology used here mainly follows Söli 1997. Terminology of some special parts of male genitalia is explained in the figures. The following acronyms for museums and collections are used in the text: ZMUT – Zoological Museum, University of Turku, Turku, Finland; CNC – Canadian National Collection of Insects, Arachnids and Nematodes, Ottawa, Canada; JES – Private collection of Jukka Salmela, Rovaniemi, Finland; TZS - Tromsø Museum, The Arctic University of Norway, Tromsø, Norway. All specimens in JES and ZMUT are stored in 70 % ethanol, kept in 2 ml plastic vials with a screw cap and a rubber o-ring seal. Hypopygia of some specimens are kept in separate 0,5 ml microvials in glycerol.

Digital photos were captured by Olympus E520 digital camera, attached to an Olympus SZX16 stereomicroscope using Deep Focus 3.1 and Quick PHOTOCAMERA 2.3 software. Layer photos were combined using Combine ZP software. Black and white drawings were produced by using Olympus BX51 microscope with a drawing tube. Measurements of the new species are given in Table 1.

We also tried to extract DNA barcode (see e.g. Mutanen et al. 2012), as most of our specimens were stored in ethanol. These were sent to the Biodiversity Institute of Ontario, University of Guelph, Canada, for DNA extraction and sequencing. Unfortunately our specimens yielded either no DNA or resulting sequences (n=2) were most likely contaminated. These two COI mtDNA fragments showed affinities to taxa that are quite unrelated to the species that they were supposed to represent, and the results are thus ignored here.

## Taxon treatments

### Boletina valteri

Salmela sp. n.

urn:lsid:zoobank.org:act:47F834BD-35BA-4898-BD06-4EDAED1D6FE0

#### Materials

- **Type status:** Holotype. **Occurrence:** catalogNumber: DIPT-JS-2014-0197; recordedBy: J. Salmela; individualCount: 1; sex: male; **Location:** country: Finland; stateProvince: Lapponia kemensis pars orientalis; verbatimLocality: Sodankylä, Pomokaira-Tenniöaapa Mire Conservation Area, Syväkuru; verbatimLatitude: 67.8717; verbatimLongitude: 26.2127; verbatimCoordinateSystem: decimal degrees; verbatimSRS: WGS84; **Event:** samplingProtocol: Malaise trap; eventDate: 2013-6-11/7-10; habitat: Salix swamp with seepages; **Record Level:** institutionCode: ZMUT

#### Description

**Figure 4.**
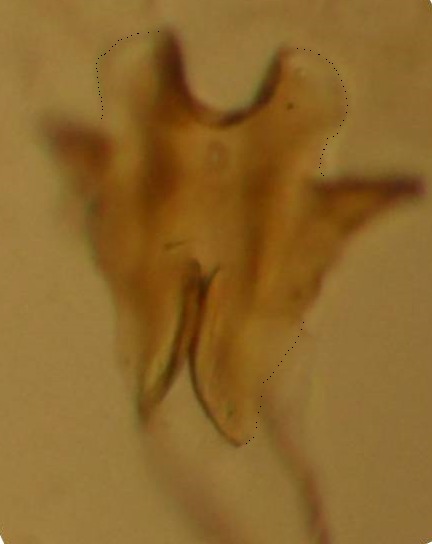
*Boletina valteri* Salmela sp.n., holotype male, a chitinized structure between submedian appendages of gonocoxites. Edges of this structure are clarified with dots.

Head dark brown–black, mouthparts brown. Palpomeres 1–3 brownish, 4–5 lighter. Scape, pedicel and flagellomeres dark-brown (six basal flagellomeres present, Fig. 2a). Scutum dark-brown, laterally brown, with scattered light setae. Pleura brown, mediotergite, laterotergite, anepimeron, anepisternum and preepisternum glabrous. Coxae, femora and tibiae yellow, trochanters infuscated, tarsomeres brown, claws black. Mid-legs missing. Wing length 2.8 mm. Sc1, Sc2, Rs, ta, tb and M-stem bare, M1, M2, CuA1, CuA2 setose on ventral surface, stem of CuA bearing a few setae distally. Sc1 ending in costa above Rs. Costa extending beyond tip of R5 to approximately 1/3 of the distance between R5 and M1 (Fig. 2a). Base of posterior fork a little beyond base of M-stem. Halteres pale. Abdominal tergites and sternites brown, bearing light hairs. 9th tergite brown. Cercus with three combs (i.e. rows of dark-brown, stout setae): proximal row with 13–15, mid row with 17–18 and distal row with 11 setae; distal setae longer than mid or proximal setae (Fig. 2b). Sternal submedian appendages of gonocoxites as in Fig. 3a; there is a an accessory structure between submedian appendages, having a distinct U-shaped median notch (Fig. 4). Lobe of gonostylus pointed, bearing two black apical setae (Fig. 3a, b, c). Parameres almost straight (apices slightly bent), rather long and thin, tapering toward apices (Fig. 3d, e, f). Aedeagus in lateral view with a blunt tip (Fig. 3f), ejaculatory apodemes as in Fig. 3d, e, f.

#### Diagnosis

A small *Boletina* species with unpatterned wings and a monochromatic abdomen. The cercus has three rows of stout setae, and the parameres are long and thin. The lobe of gonostylus is pointed. The new species is externally somewhat similar to *B. pallidula* Edwards, but that species has two rows of setae on cercus and shorter inner branch of gonostylus.

#### Etymology

The species is named after Valter Keltikangas (1905-1990), Finnish forest researcher and professor. In his famous novel "*Seitsemän tuntia erämaata*" (1977) he recollected his expedition to the vast mires and forests of Pomokaira, the type locality of the new species. The name of the new species is a genitive.

#### Distribution

European, so far only known from central Finnish Lapland.

#### Ecology

The type locality is a *Salix* swamp with meso-eutrophic groundwater seepages, surrounded by an old-growth boreal forest, dominated by spruce (Picea abies ssp. obovata), birch (*Betula* sp.), and bilberry (*Vaccinium myrtillus*) on the ground layer.

#### Taxon discussion

The new species seems to be quite isolated from other known species of the genus. It has relatively short antennal flagellomeres (length:width ratio of the 2nd flagellomere is 2), long and straight parameres and inconspicuous parameral apodemes. The parameres are also moveable in their bases. The basal, paired projections of the aedeagal complex are here considered as ejaculatory apodemes. Even more striking is the presence of an accessory structure lying close to sternal submedian appendages of gonocoxites. This structure is perhaps a part of aedeagal complex, a highly specialized organ derived from ventrodistal sclerites of aedeagus. The new species is somewhat similar to *B. pallidula*, but in addition to the diffrences in male genitalia given above, *B. pallidula* has lighter body coloration, slightly longer flagellomeres and is lacking vein Sc2.

### Boletina kullervoi

Salmela sp. n.

urn:lsid:zoobank.org:act:9ECA0B70-AB7E-4E3D-92EA-876ED14CB061

#### Materials

- **Type status:** Holotype. **Occurrence:** catalogNumber: MYCE-JS-2013-0148; recordedBy: J. Salmela; individualCount: 1; sex: male; **Location:** country: Finland; stateProvince: Lapponia kemensis pars orientalis; verbatimLocality: Savukoski, Törmäoja; verbatimLatitude: 67.8468; verbatimLongitude: 29.4724; verbatimCoordinateSystem: decimal degrees; verbatimSRS: WGS84; **Event:** samplingProtocol: Malaise trap; eventDate: 2012-8-16/9-18; habitat: spring, young deciduous forest; **Record Level:** institutionCode: ZMUT
- **Type status:** Paratype. **Occurrence:** catalogNumber: DIPT-JS-2014-0109; recordedBy: J. Salmela; individualCount: 1; sex: male; **Location:** country: Finland; stateProvince: Regio kuusamoensis; verbatimLocality: Salla, Värriö Strict Nature Reserve, Kuntasjoki; verbatimLatitude: 67.7494; verbatimLongitude: 29.6168; verbatimCoordinateSystem: decimal degrees; verbatimSRS: WGS84; **Event:** samplingProtocol: Malaise trap; eventDate: 2013-7-29/9-19; habitat: headwater stream, old-growth boreal forest; **Record Level:** institutionCode: JES
- **Type status:** Paratype. **Occurrence:** catalogNumber: DIPT-JS-2015-0341; recordedBy: J. Salmela; individualCount: 1; sex: male; **Location:** country: Finland; stateProvince: Lapponia kemensis pars orientalis; verbatimLocality: Savukoski, Törmäoja; verbatimLatitude: 67.8468; verbatimLongitude: 29.4724; verbatimCoordinateSystem: decimal degrees; verbatimSRS: WGS84; **Event:** samplingProtocol: Malaise trap; eventDate: 2012-8-16/9-18; habitat: spring, young deciduous forest; **Record Level:** institutionCode: JES

#### Description

Head black, mouthparts dark-brown (Fig. 5). Palpomeres 1–3 dark-brown, 4–5 lighter, but apex of palpomere 5 darkened. Scape, pedicel and flagellomeres dark-brown, except base of 1st flagellomere light brown (see Table 1). Scutum dark-brown, with scattered light setae. Pleura dark brown, mediotergite, laterotergite, anepimeron, anepisternum and preepisternum glabrous. Fore and mid coxae, all femora and tibiae yellow, trochanters black, tarsomeres brown, claws black; basal half of hind coxa dark brown. Wing length 4.8 mm. Sc1, Sc2, Rs, ta, tb and M-stem bare, M1, M2, CuA1, CuA2 and stem of CuA setose on ventral surface. Sc1 ending in costa little beyond Rs. Costa extending beyond tip of R5 only slightly (Fig. 5a). Halteres pale yellow. Abdominal tergites and sternites brown, bearing light hairs. 9th tergite dark-brown. Cercus ca. 1.2 times wider than long, with four more or less regular combs (i.e. rows of dark-brown, stout setae): proximal row with 5, second row with 14–15, third row 8–11 and distal row with 11–13 setae; setae of distal row a little longer than setae of proximal row (Fig. 6b). Apices of sternal submedian appendages of gonocoxites club-shaped, rounded (Fig. 6d, e, see Notes below). Inner branch of gonostylus rather long and narrow, bearing a black apical spine and a longer hyaline seta (Fig. 6c). Parameres short and straight (Fig. 6d, e, f). Parameral apodemes with lateral lobes (Fig. 6d). Aedeagus narrow, its apex curved (Fig. 6f). Ejaculatory apodeme with basal projection and paired ventrodistal sclerites.

#### Diagnosis

Medium-sized *Boletina* which is externally similar to *B. borealis* Zetterstedt, wings unpatterned. The new species has small, paired ventrodistal sclerites on the aedeagus, whereas *B. borealis* has larger sclerites. Parameres are short and widely separated, parameral apodemes having distinct lateral lobes.

#### Etymology

The species is named after Kullervo, the son of Kalervo. Kullervo is a tragic character in *Kalevala*, the Finnish national epic. *Kullervo* is also the first symphony composed by Jean Sibelius (1892). The name of the new species is a genitive.

#### Distribution

European, so far only known from eastern Finnish Lapland.

#### Ecology

Both collecting sites are pristine boreal forests. The collecting site in Törmäoja was a stream valley with lush vegetation, groundwater seepages and coniferous forest on the valley slopes. At Värriö the habitat was similar with the valley surrounded by sparse pine and spruce forest.

#### Taxon discussion

The new species appears to be closest to *B. borealis* and related species, including *B. intermedia* Lundström and *B. birulai* Lundström. However, the new species is readily distinguished from these based on differences in male hypopygium. Sternal submedian appendages of *B. kullervoi* sp.n. are apically club-shaped, but more truncated among *B. borealis* and other species. Parameres of *B. kullervoi* sp.n. are short and widely separated, including lateral wing-like lobes on the parameral apodemes. Parameres of *B. borealis* and other species are longer, having no such lateral projections. Please also see Notes below.

#### Notes

*Boletina kullervoi* sp.n. is characterized by the arching apices of the sternal submedian appendages of the gonocoxites. In ventral view the sternal submedian appendages appear to be truncated and slightly oblique, but in reality the apices are club-like, firmly attached to aedeagal complex. If one is trying to remove the aedeagal complex from the hypopygium, apices of sternal submedian appendages remain attached to the aedeagal complex. The ventrodistal sclerites of the ejaculatory apodeme are here termed as paired, because there are short apical and basal projections. However, further morphological study is needed to verify whether these appendages are homologous to similar structures among species such as *B. borealis* and *B. hyperborea* sp.n.

### Boletina hyperborea

Salmela sp. n.

urn:lsid:zoobank.org:act:20DAEB55-9D8B-489B-BAF3-E58F79792EF4

#### Materials

- **Type status:** Holotype. **Occurrence:** catalogNumber: MYCE-JS-2013-0185; recordedBy: J. Salmela; individualCount: 1; sex: male; **Location:** country: Finland; stateProvince: Lapponia kemensis pars orientalis; verbatimLocality: Savukoski, Joutenoja; verbatimLatitude: 67.8179; verbatimLongitude: 29.4458; verbatimCoordinateSystem: decimal degrees; verbatimSRS: WGS84; **Event:** samplingProtocol: Malaise trap; eventDate: 2012-6-14/7-10; habitat: headwater stream, boreal forest; **Record Level:** institutionCode: ZMUT
- **Type status:** Paratype. **Occurrence:** catalogNumber: DIPT-JS-2014-0014; recordedBy: J. Salmela; individualCount: 1; sex: male; **Location:** country: Finland; stateProvince: Regio kuusamoensis; verbatimLocality: Salla, Värriö Strict Nature Reserve, Kuntasjoki; verbatimLatitude: 67.7494; verbatimLongitude: 29.6168; verbatimCoordinateSystem: decimal degrees; verbatimSRS: WGS84; **Event:** samplingProtocol: Malaise trap; eventDate: 2013-7-29/9-19; habitat: headwater stream, old-growth boreal forest; **Record Level:** institutionCode: JES
- **Type status:** Paratype. **Occurrence:** catalogNumber: DIPT-JS-2014-0019; recordedBy: J. Salmela; individualCount: 1; sex: male; **Location:** country: Finland; stateProvince: Regio kuusamoensis; verbatimLocality: Salla, Värriö Strict Nature Reserve, Kuntasjoki; verbatimLatitude: 67.7494; verbatimLongitude: 29.6168; verbatimCoordinateSystem: decimal degrees; verbatimSRS: WGS84; **Event:** samplingProtocol: Malaise trap; eventDate: 2013-7-29/9-19; habitat: headwater stream, old-growth boreal forest; **Record Level:** institutionCode: JES
- **Type status:** Paratype. **Occurrence:** catalogNumber: DIPT-JS-2014-0485; recordedBy: J. Salmela; individualCount: 1; sex: male; **Location:** country: Finland; stateProvince: Regio kuusamoensis; verbatimLocality: Salla, Värriö Strict Nature Reserve, Kuntasjoki; verbatimLatitude: 67.7494; verbatimLongitude: 29.6168; verbatimCoordinateSystem: decimal degrees; verbatimSRS: WGS84; **Event:** samplingProtocol: Malaise trap; eventDate: 2013-7-29/9-19; habitat: headwater stream, old-growth boreal forest; **Record Level:** institutionCode: JES
- **Type status:** Paratype. **Occurrence:** catalogNumber: DIPT-JS-2015-0003; recordedBy: J. Salmela; individualCount: 1; sex: male; **Location:** country: Finland; stateProvince: Lapponia enontekiensis; verbatimLocality: Enontekiö, Käsivarsi Wilderness Area, Toskaljärvi N; verbatimLatitude: 69.1986; verbatimLongitude: 21.4530; verbatimCoordinateSystem: decimal degrees; verbatimSRS: WGS84; **Event:** samplingProtocol: Malaise trap; eventDate: 2014-7-2/8-21; habitat: rich, subalpine sloping fen, seepages; **Record Level:** institutionCode: ZMUT
- **Type status:** Paratype. **Occurrence:** recordedBy: P.J. Skitsko; individualCount: 1; sex: male; **Location:** country: Canada; stateProvince: Yukon; verbatimLocality: Ogilvie Mountains, NE of Dawson City, North Fork Crossing, mile 43, Peel Plateau Rd.; verbatimElevation: 3500 ft; verbatimLatitude: 64.433; verbatimLongitude: -138.233; verbatimCoordinateSystem: decimal degrees; verbatimSRS: WGS84; **Event:** eventDate: 1962-7-4; **Record Level:** institutionCode: CNC
- **Type status:** Other material. **Occurrence:** recordedBy: P.J. Skitsko; individualCount: 7; sex: male; **Taxon:** genus: Boletina; specificEpithet: hyperborea; scientificNameAuthorship: Salmela; **Location:** country: Canada; stateProvince: Yukon; verbatimLocality: Ogilvie Mountains, NE of Dawson City, North Fork Crossing, mile 43, Peel Plateau Rd.; verbatimElevation: 3500 ft; verbatimLatitude: 64.433; verbatimLongitude: -138.233; verbatimCoordinateSystem: decimal degrees; verbatimSRS: WGS84; **Event:** eventDate: 1962-7-4; **Record Level:** institutionCode: CNC
- **Type status:** Other material. **Occurrence:** recordedBy: P.J. Skitsko; individualCount: 1; sex: male; **Taxon:** genus: Boletina; specificEpithet: hyperborea; scientificNameAuthorship: Salmela; **Location:** country: Canada; stateProvince: Yukon; verbatimLocality: Ogilvie Mountains, NE of Dawson City, North Fork Crossing, mile 42, Peel Plateau Rd.; verbatimElevation: 3500 ft; verbatimCoordinateSystem: decimal degrees; verbatimSRS: WGS84; **Event:** eventDate: 1962-6-24; **Record Level:** institutionCode: CNC
- **Type status:** Other material. **Occurrence:** recordedBy: R.E. Leech; individualCount: 2; sex: male; **Taxon:** genus: Boletina; specificEpithet: hyperborea; scientificNameAuthorship: Salmela; **Location:** country: Canada; stateProvince: Yukon; verbatimLocality: Ogilvie Mountains, NE of Dawson City, North Fork Crossing, mile 43, Peel Plateau Rd.; verbatimElevation: 3500 ft; verbatimLatitude: 64.433; verbatimLongitude: -138.233; verbatimCoordinateSystem: decimal degrees; verbatimSRS: WGS84; **Event:** eventDate: 1962-7-4; **Record Level:** institutionCode: CNC
- **Type status:** Other material. **Occurrence:** recordedBy: P.J. Skitsko; individualCount: 2; sex: male; **Taxon:** genus: Boletina; specificEpithet: hyperborea; scientificNameAuthorship: Salmela; **Location:** country: Canada; stateProvince: Yukon; verbatimLocality: Ogilvie Mountains, NE of Dawson City, North Fork Crossing; **Event:** eventDate: 1962-7-3; **Record Level:** institutionCode: CNC
- **Type status:** Other material. **Occurrence:** recordedBy: J. Kjaerandsen; individualCount: 2; sex: 1 male, 1 female; **Taxon:** genus: Boletina; specificEpithet: hyperborea; scientificNameAuthorship: Salmela; **Location:** country: Finland; stateProvince: Lapponia inarensis; verbatimLocality: Utsjoki, Kevo SNR; verbatimLatitude: 69.7238; verbatimLongitude: 27.0428; verbatimCoordinateSystem: decimal degrees; verbatimSRS: WGS84; **Identification:** identifiedBy: J. Kjaerandsen; **Event:** eventDate: 2014-7-4/17; **Record Level:** collectionCode: TZS
- **Type status:** Other material. **Occurrence:** recordedBy: T. E. Barstad; individualCount: 1; sex: male; **Taxon:** genus: Boletina; specificEpithet: hyperborea; scientificNameAuthorship: Salmela; **Location:** country: Norway; stateProvince: Finnmark; verbatimLocality: Vardø, Komagdalen, Stuorra Suovka; verbatimLatitude: 70.1; verbatimLongitude: 29.7; verbatimCoordinateSystem: decimal degrees; verbatimSRS: WGS84; **Identification:** identifiedBy: J. Kjaerandsen; **Event:** eventDate: 2009-7-2/15; **Record Level:** collectionCode: TZS
- **Type status:** Other material. **Occurrence:** recordedBy: T. E. Barstad; individualCount: 8; sex: male; **Taxon:** genus: Boletina; specificEpithet: hyperborea; scientificNameAuthorship: Salmela; **Location:** country: Norway; stateProvince: Finnmark; verbatimLocality: Båtsfjord, Komagdalen, Bajit Suovka; verbatimLatitude: 70.1; verbatimLongitude: 29.7; verbatimCoordinateSystem: decimal degrees; verbatimSRS: WGS84; **Identification:** identifiedBy: J. Kjaerandsen; **Event:** eventDate: 2009-7-14/8-20; **Record Level:** collectionCode: TZS
- **Type status:** Other material. **Occurrence:** recordedBy: T. E. Barstad; individualCount: 1; sex: male; **Taxon:** genus: Boletina; specificEpithet: hyperborea; scientificNameAuthorship: Salmela; **Location:** country: Norway; stateProvince: Finnmark; verbatimLocality: Komagdalen, Bjørnskaret; verbatimLatitude: 70.1; verbatimLongitude: 29.7; verbatimCoordinateSystem: decimal degrees; verbatimSRS: WGS84; **Identification:** identifiedBy: J. Kjaerandsen; **Event:** eventDate: 2009-7-1/14; **Record Level:** collectionCode: TZS
- **Type status:** Other material. **Occurrence:** recordedBy: K. Müller; individualCount: 1; sex: male; **Taxon:** genus: Boletina; specificEpithet: hyperborea; scientificNameAuthorship: Salmela; **Location:** country: Sweden; stateProvince: Torne Lappmark; verbatimLocality: Kiruna, Abisko; verbatimLatitude: 68.3; verbatimLongitude: 18.8; verbatimCoordinateSystem: decimal degrees; verbatimSRS: WGS84; **Identification:** identifiedBy: J. Kjaerandsen; **Event:** eventDate: 1976-6-23/30; **Record Level:** collectionCode: TZS

#### Description

**Figure 7.**
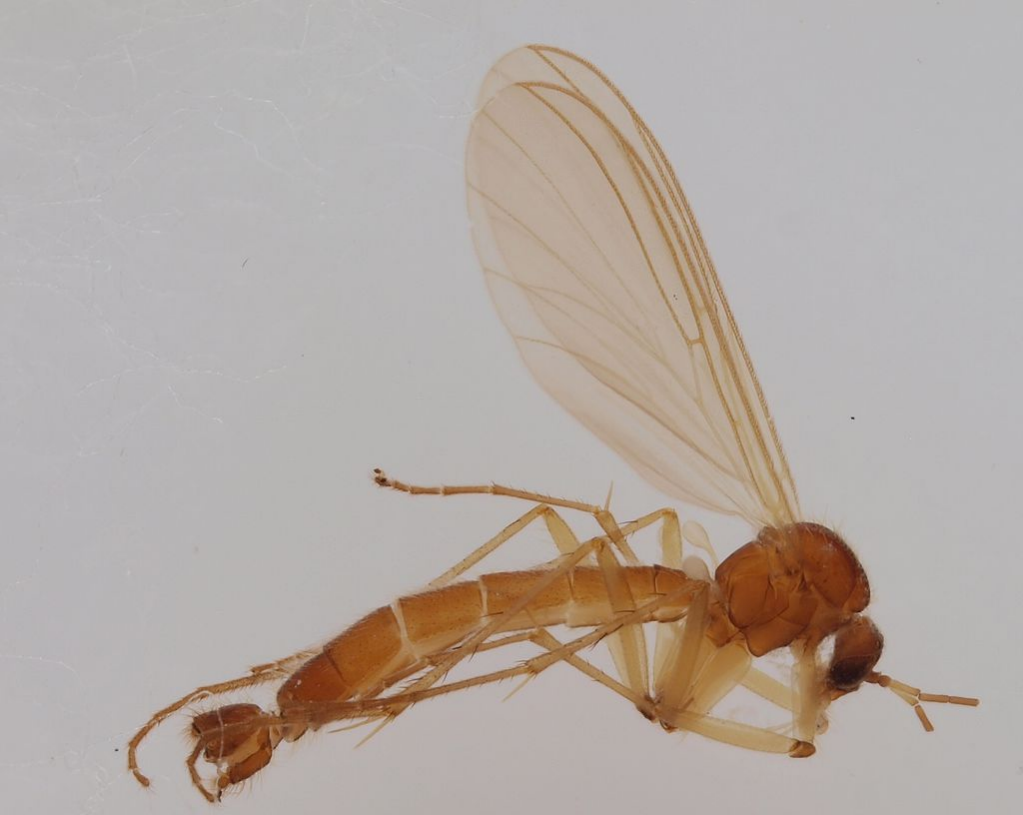
*Boletina hyperborea* Salmela sp.n., paratype male DIPT-JS-2014-0485, habitus, lateral view.

Head dark brown, mouthparts brown. Palpomeres light-brown. Scape and pedicel dark-brown, 1st flagellomere light brown, other flagellomeres brown. Scutum brown, rather densely covered by light setae. Pleura brown, mediotergite, laterotergite, anepimeron, anepisternum and preepisternum glabrous. Coxae, femora and tibiae yellow, trochanters infuscated, tarsomeres brown, claws black. Wing length 4.5 mm. Sc1, Sc2, Rs, ta, tb and M-stem bare, M1, M2, CuA1, CuA2 setose on ventral surface, stem of CuA bearing a few setae distally. Sc1 ending in costa slightly before Rs. Costa extending beyond tip of R5 only slightly (Fig. 7). Halteres pale. Abdominal tergites and sternites brown, bearing light hairs. 9th tergite brown, covered by long brownish setae. Cercus ca. 1.7 times wider than long, with five more or less regular combs (i.e. rows of dark-brown, equally long stout setae), total number of setae ca. 120–130 (Fig. 8b). Sternal submedian appendages of gonocoxites apically truncated, rather narrow and bulging medially (Fig. 8a). Inner branch of gonostylus long and narrow, pointed, bearing a black apical spine. Aedeagal complex rather long and narrow (Fig. 8c, e). Parameres long and thin, not exceeding tip of aedeagus, apically incurved (Fig. 8c, d, f). Basal part of parameres dark, apices hyaline. Parameral apodemes stout, in lateral view widest medially (Fig. 8c, d). Ejaculatory apodeme basally bilobed (Fig. 8e). Aedeagus narrowing apically, having a hyaline membrane around the apex. Aedeagus with bare or non-spinose ventrodistal sclerites, serving as attachment points to sternal submedian appendages of gonocoxites.

#### Diagnosis

Medium-sized *Boletina* belonging to a group of species around *B. borealis* Zetterstedt. Wings and abdomen unpatterned. Sternal submedian appendages of gonocoxites bulging medially. Parameres rather long and thin, apically incurved, bare. Parameral apodemes are wide in lateral view and the aedeagus is coated by a transparent membrane. The species is superficially similar to *B. hymenophalloides* Sasakawa & Kimura; that species is characterized by 1) wide and apically spinulose parameres, their apices reaching the tip of the aedeagus, 2) a more narrow aedeagus and 3) a more extensive membrane around the aedeagus. It is most likely that *B. hymenophalloides* is not closely related to *B. borealis* and similar-looking species.

#### Etymology

Hyperborea (an noun in apposition) is taken from the Greek mythology, meaning the far north.

#### Distribution

Northern parts of Fennoscandia (Finland, Norway, Sweden) and Canada. In Fennoscandia the species have been observed from the northern boreal zone and from the subarctic ecoregion. In Canada it has been observed from the Ogilvie Mountains in the Yukon territory, which is in the subarctic ecoregion.

#### Ecology

The collecting sites are boreal forests and alpine wetlands. Joutenoja and Värriö are headwater streams, characterized by groundwater seepages and surrounded by coniferous forests. Toskaljärvi is an oroarctic, alpine sloping rich-fen, dominated by brown mosses.

### Boletina nuortti

Salmela sp. n.

urn:lsid:zoobank.org:act:41EC45C7-F933-4715-A296-0FD7CD91D991

#### Materials

- **Type status:** Holotype. **Occurrence:** catalogNumber: DIPT-JS-2015-0074; recordedBy: J. Salmela; individualCount: 1; sex: male; **Location:** country: Finland; stateProvince: Lapponia kemensis pars orientalis; verbatimLocality: Savukoski, Hannu Ollin vaara; verbatimLatitude: 67.8439; verbatimLongitude: 29.4685; verbatimCoordinateSystem: decimal degrees; verbatimSRS: WGS84; **Event:** samplingProtocol: Malaise trap; eventDate: 2013-8-7/9-19; habitat: old-growth boreal forest, dominated by pine and birch; **Record Level:** institutionCode: ZMUT
- **Type status:** Paratype. **Occurrence:** catalogNumber: DIPT-JS-2015-0159; recordedBy: J. Salmela; individualCount: 1; sex: male; **Location:** country: Finland; stateProvince: Lapponia kemensis pars orientalis; verbatimLocality: Savukoski, Hannu Ollin vaara; verbatimLatitude: 67.8439; verbatimLongitude: 29.4685; verbatimCoordinateSystem: decimal degrees; verbatimSRS: WGS84; **Event:** samplingProtocol: Malaise trap; eventDate: 2013-8-7/9-19; habitat: old-growth boreal forest, dominated by pine and birch; **Record Level:** institutionCode: JES

#### Description

Head dark brown, mouthparts brown and palpomeres brown. Scape and pedicel dark-brown, flagellomeres brown. Scutum brown, rather densely covered by light setae. Pleura brown, mediotergite, laterotergite, anepimeron, anepisternum and preepisternum glabrous. Coxae, femora and tibiae yellow, trochanters infuscated, tarsomeres light-brown, claws black. Wing length 3.4 mm. Sc1, Sc2, Rs, ta, tb, CuA and M-stem bare, apical halves of M1, M2, CuA1 and CuA2 setose on ventral surface. Sc1 ending in costa before Rs. Costa extending beyond tip of R5 to approximately 1/3 of the distance between R5 and M1. Halteres pale. Abdominal tergites and sternites brown, bearing dark hairs. 9th tergite brown, covered by brownish setae. Cercus ca. 1.75 times wider than long, with ca. four irregular combs (i.e. rows of dark-brown, equally long stout setae), total number of setae ca. 50–60 (Fig. 9b). Sternal submedian appendages of gonocoxites apically rounded, short and separated by a Y-shaped cleft (Fig. 9a). Gonostylus appearing unibranched, but is divided on inner and outer branches (Fig. 10a, b). Outer branch rather inconspicuous, bearing thin and rather short mesal hairs. Inner branch short, mostly glabrous, with a few small subapical setae, two or three black apical spines and a longer hyaline seta. Aedeagal complex rather short and wide (Fig. 10c, d, e). Parameres monochromatic, short and stout, tapering apically, their apices not exceeding tip of aedeagus (Fig. 10d). Parameral apodemes very wide in lateral view (Fig. 10e). Ejaculatory apodeme consisting of two short and wide basal lobes. Aedeagus with short ventrodistal sclerites, apex of aedeagus down-curved, coated by a hyaline membrane (Fig. 10c, e).

#### Diagnosis

A medium-sized *Boletina* species similar to *B. silvatica* Dziedzicki, amongst other species. Apices of the sternal submedian appendages are small, rounded and separated by a Y-shaped cleft. The new species is lacking dorsal projections on the gonostylus, as typical for species such as *B. silvatica*, *B. subtriangularis* Polevoi & Hedmark and *B. triangularis* Polevoi. Parameres of the new species are also distinctly shorter than those of the three above mentioned species. Wings unpatterned.

#### Etymology

The name of the new species is derived from the River Nuortti, a large tributary of the River Tuuloma. Nuortti is derived from north Sami word (nuorti), meaning east; the River Nuortti flows in a NE direction from Finland to Russia. The name of the new species is a noun in apposition.

#### Distribution

European, so far only known from Törmäoja conservation area, eastern Finnish Lapland.

#### Ecology

The type locality is a birch dominated boreal forest, bilberry and cowberry (*Vaccinium vitis-idaea*) on the ground layer.

#### Taxon discussion

The new species is reminiscent of *B. silvatica* and related species, especially the shape of the aedeagus which indicates an affinity to *B. nuortti* sp.n. within this species group. However, species related to *B. silvatica* have a very deep sternal cleft of gonocoxites, whereas the cleft in *B. nuortti* sp.n. is not exceeding the half length of the gonocoxites. In addition, *B. silvatica* and related species possess a dorsal projection on gonostylus, a character absent on *B. nuortti* sp.n. Thirdly, parameres of the new species are relatively short; parameres of *B. silvatica* and related species greatly exceed the length of aedeagus (see e.g. Polevoi and Hedmark 2004).

### Boletina arctica

Holmgren, 1872

Boletina arctica Holmgren, 1872: 105 (Holmgren 1872)
Boletina arctica var. Edwards, 1933: 612 (Edwards 1933)
Boletina arctica nec Rübsaamen, 1898: 104 (Rübsaamen 1898), (=*Boletina digitata* Lundström)

#### Materials

- **Type status:** Other material. **Occurrence:** recordedBy: H.K. Rutz; individualCount: 3; sex: male; **Location:** country: Canada; verbatimLocality: Axel Heiberg Island, Wh. Glacier; verbatimLatitude: 79.416; verbatimLongitude: -90.750; verbatimCoordinateSystem: WGS84; **Event:** eventDate: 1963-7-26; **Record Level:** institutionCode: CNC
- **Type status:** Other material. **Occurrence:** recordedBy: H.K. Rutz; individualCount: 4; sex: male; **Location:** country: Canada; verbatimLocality: Axel Heiberg Island, Wolf R.; verbatimLatitude: 79.416; verbatimLongitude: -90.750; verbatimCoordinateSystem: WGS84; **Event:** eventDate: 1963-7-25; **Record Level:** institutionCode: CNC
- **Type status:** Other material. **Occurrence:** recordedBy: H.K. Rutz; individualCount: 1; sex: male; **Location:** country: Canada; verbatimLocality: Axel Heiberg Island, Wolf R.; verbatimLatitude: 79.416; verbatimLongitude: -90.750; verbatimCoordinateSystem: WGS84; **Event:** eventDate: 1963-7-24; **Record Level:** institutionCode: CNC
- **Type status:** Other material. **Occurrence:** recordedBy: H.K. Rutz; individualCount: 3; sex: female; **Location:** country: Canada; verbatimLocality: Axel Heiberg Island, Wolf R.; verbatimLatitude: 79.416; verbatimLongitude: -90.750; verbatimCoordinateSystem: WGS84; **Event:** eventDate: 1963-7-25; **Record Level:** institutionCode: CNC
- **Type status:** Other material. **Occurrence:** recordedBy: H.K. Rutz; individualCount: 1; sex: female; **Location:** country: Canada; verbatimLocality: Axel Heiberg Island, Wh. Glacier; verbatimLatitude: 79.416; verbatimLongitude: -90.750; verbatimCoordinateSystem: WGS84; **Event:** eventDate: 1963-7-26; **Record Level:** institutionCode: CNC
- **Type status:** Other material. **Occurrence:** recordedBy: H.K. Rutz; individualCount: 1; sex: female; **Location:** country: Canada; verbatimLocality: Axel Heiberg Island; verbatimLatitude: 79.416; verbatimLongitude: -90.750; verbatimCoordinateSystem: WGS84; **Event:** eventDate: 1963-8-4; **Record Level:** institutionCode: CNC

#### Description

Redescription

Male: Head, mouthparts and palpomeres black. Scape, pedicel and flagellomeres dark-brown – black. Scutum dark-brown, with scattered light setae. Pleura dark brown, mediotergite, laterotergite, anepimeron, anepisternum and preepisternum glabrous. Fore coxae, all femora and tibiae yellow, trochanters black, tarsomeres brown, claws black. Mid and hind coxae dark-brown. Wing length 4.8 mm. Sc1, Sc2, Rs, ta, tb and M-stem bare, apical halves of M1, M2 and CuA1, entire CuA2 and stem of CuA setose on dorsal surface. Sc1 ending in costa little before Rs. Costa extending beyond tip of R5 to approximately 1/3 of the distance between R5 and M1. Halteres pale yellow. Abdominal tergites and sternites dark-brown, bearing light hairs. 9th tergite black. Cercus ca. 1.4 times wider than long, having basally 3–4 irregular combs (i.e. rows of dark-brown, stout setae): total number of such setae 37–40. Apex of cercus with a distinct comb, number of setae 14–15. Sternal submedian appendages of gonocoxites short, apically rounded, diverging, pubescent (Fig. 11c). Gonocoxite apicomesally with two black spines (Fig. 11a). Inner branch of gonostylus pollex-like, bearing two long black apical setae and a black spine. Outer branch of gonostylus rectangular, widest subapically, with a modest apical peak (Fig. 11a, b). Mesal edge of gonostylus hairy. Parameres short and straight (Fig. 11c). Parameral apodemes + parameres resembling the letter L in lateral view (Fig. 11d). Aedeagus short and curved (Fig. 11d). Ejaculatory apodeme with basal projections. Gonocoxite internally with a narrow, pointed projection (Fig. 11a). Close to this projection are two hyaline projections, bearing weak bristles; this latter projection is part of aedeagal complex.

#### Distribution

*Boletina arctica* has been recorded from Greenland (Holmgren 1872, Söli et al. 2015), Arctic Russia (Zaitzev 1994) and Akpatok Island, Hudson Strait, low arctic zone of Canada (Edwards 1933). Here we report the species from the high arctic zone, Axel Heiberg Island, Canada.

#### Taxon discussion

Given the rarity of this species, *B. arctica* has been illustrated rather frequently in taxonomic literature. The first to illustrate this species was Rübsaamen (Rübsaamen 1898), but instead of *B. arctica*, he actually figured *B. digitata* Lundström (or some related species of this complex, see Suppl. material 1). Johannsen (Johannsen 1911) copied Rübsaamen's illustration of *B. arctica* and gave no additional occurrence data. Later Lundström (Lundström 1912) provided good figures, based on material loaned from ZMUC, Copenhagen, originally collected from Greenland. It is not clear whether a type specimen was examined, but most likely it was a non-type male, since the type material of *B. arctica* is not deposited in ZMUC (http://www.zmuc.dk/EntoWeb/collections-databaser/Diptera/Mycetophilidae%20all.htm). Hence, authors after Lundström have followed his interpretation of *B. arctica*. Edwards (Edwards 1933) was actually the first to report this species from North America (Akpatok Island), he illustrated *Boletina arctica* var. According to Edwards, his specimens were slightly different from a male specimen collected from Greenland. However, such differences on the outline of the gonostylus are due to aspect of the gonostylus to the viewer (Fig. 11b). *Boletina arctica* was also illustrated by Zaitzev (Zaitzev 1994) and recently by Söli et al. (Söli et al. 2015).

The species possesses some unique characters that have been misinterpreted in the literature. First of all, *B. arctica* has no sharply pointed parameres (cf. Edwards 1933​, p. 612). Lundström, Edwards and Zaitzev have illustrated these sharply-pointed projections that actually stem from the inner lateral margin of the gonocoxite. Furthermore, close to these projections lie two spinose, hyaline projections that orginate from the aedeagal complex. The parameres themselves are inconspicuous, short and straight.

### Boletina borealis

Zetterstedt, 1852

Boletina borealis Zetterstedt, 1852: 4160 (Zetterstedt 1852)
Boletina borealis syn. *Boletina tundrica* Dziedzicki, 1915: 1 (Becker et al. 1915)

#### Materials

- **Type status:** Other material. **Occurrence:** recordedBy: R.B. Madge; individualCount: 1; sex: male; **Location:** country: Canada; verbatimLocality: Ellesmere Island, Quttinirpaaq National Park, Hazen Camp; verbatimLatitude: 81.490; verbatimLongitude: -71.180; verbatimCoordinateSystem: decimal degrees; verbatimSRS: WGS84; **Identification:** identifiedBy: J. Salmela; **Event:** eventDate: 1962-7-1; **Record Level:** institutionCode: CNC
- **Type status:** Other material. **Occurrence:** recordedBy: J.R. Vockeroth; individualCount: 1; sex: male; **Location:** country: Canada; verbatimLocality: North West Territories, Chesterfield; **Identification:** identifiedBy: J. Salmela; **Event:** eventDate: 1950-7-23; **Record Level:** institutionCode: CNC
- **Type status:** Other material. **Occurrence:** recordedBy: E.E. MacDougall; individualCount: 1; sex: male; **Location:** country: Canada; verbatimLocality: British Columbia, Toad river, Alaska Hwy, Mi440; maximumElevationInMeters: 1370; **Identification:** identifiedBy: J. Salmela; **Event:** eventDate: 1959-6-19; **Record Level:** institutionCode: CNC
- **Type status:** Other material. **Occurrence:** recordedBy: E.H.N Smith; individualCount: 1; sex: male; **Location:** country: Canada; verbatimLocality: North West Territories, Cambridge bay; **Identification:** identifiedBy: J. Salmela; **Event:** eventDate: 1950-7-11; **Record Level:** institutionCode: CNC
- **Type status:** Other material. **Occurrence:** recordedBy: J.G. Chillcott; individualCount: 1; sex: male; **Location:** country: Canada; verbatimLocality: North West Territories, Spence Bay; **Identification:** identifiedBy: J. Salmela; **Event:** eventDate: 1951-7-14; **Record Level:** institutionCode: CNC

#### Distribution

Holarctic, here reported for the first time from the Nearctic region. The species is known from North Europe, Central European mountains (see Jakovlev et al. 2014 and references therein) and Japan (Hokkaido, Sasakawa and Kimura 1974​).

### Boletina birulai

Lundström, 1915

Boletina birulai Lundström, 1915: 3 (Lundström 1915)

#### Materials

- **Type status:** Other material. **Occurrence:** recordedBy: R.E. Leech; individualCount: 8; sex: male; **Location:** country: Canada; verbatimLocality: Yukon, Nort Fork Crossing, Mi. 43 Peel plt.Rd; minimumElevationInMeters: 1060; **Identification:** identifiedBy: J. Salmela; **Event:** eventDate: 1962-7-4; **Record Level:** institutionCode: CNC
- **Type status:** Other material. **Occurrence:** recordedBy: R.E. Leech; individualCount: 2; sex: male; **Location:** country: Canada; verbatimLocality: Yukon, Nort Fork Crossing, Mi. 43 Peel plt.Rd; minimumElevationInMeters: 1060; **Identification:** identifiedBy: J. Salmela; **Event:** eventDate: 1962-7-5; **Record Level:** institutionCode: CNC
- **Type status:** Other material. **Occurrence:** recordedBy: R.E. Leech; individualCount: 1; sex: male; **Location:** country: Canada; verbatimLocality: Yukon, Nort Fork Crossing, Mi. 43 Peel plt.Rd; minimumElevationInMeters: 1060; **Identification:** identifiedBy: J. Salmela; **Event:** eventDate: 1962-7-6; **Record Level:** institutionCode: CNC
- **Type status:** Other material. **Occurrence:** recordedBy: P.J. Skitsko; individualCount: 5; sex: male; **Location:** country: Canada; verbatimLocality: Yukon, Nort Fork Crossing, Mi. 43 Peel plt.Rd; minimumElevationInMeters: 1060; **Identification:** identifiedBy: J. Salmela; **Event:** eventDate: 1962-7-4; **Record Level:** institutionCode: CNC
- **Type status:** Other material. **Occurrence:** recordedBy: G. & D.M. Wood; individualCount: 2; sex: male; **Location:** country: Canada; verbatimLocality: Yukon, Dempster Highway, Mi. 51; **Identification:** identifiedBy: J. Salmela; **Event:** eventDate: 1973-6-25/27; **Record Level:** institutionCode: CNC
- **Type status:** Other material. **Occurrence:** recordedBy: W.R.M. Mason; individualCount: 1; sex: male; **Location:** country: Canada; verbatimLocality: Yukon, Herschel Island; **Identification:** identifiedBy: J. Salmela; **Event:** eventDate: 1971-7-1; **Record Level:** institutionCode: CNC
- **Type status:** Other material. **Occurrence:** recordedBy: D.M. Wood; individualCount: 1; sex: male; **Location:** country: Canada; verbatimLocality: Yukon, Herschel Island; **Identification:** identifiedBy: J. Salmela; **Event:** eventDate: 1971-6-28/29; **Record Level:** institutionCode: CNC
- **Type status:** Other material. **Occurrence:** recordedBy: P.J. Skitsko; individualCount: 1; sex: male; **Location:** country: Canada; verbatimLocality: Yukon, North Fork Pass, Ogilvie Mts.; minimumElevationInMeters: 1200; **Identification:** identifiedBy: J. Salmela; **Event:** eventDate: 1962-6-18; **Record Level:** institutionCode: CNC
- **Type status:** Other material. **Occurrence:** recordedBy: B.S. Heming; individualCount: 1; sex: male; **Location:** country: USA; verbatimLocality: Alaska, Cape Thompson; **Identification:** identifiedBy: J. Salmela; **Event:** eventDate: 1961-7-26; **Record Level:** institutionCode: CNC
- **Type status:** Other material. **Occurrence:** recordedBy: B.S. Heming; individualCount: 1; sex: male; **Location:** country: USA; verbatimLocality: Alaska, Cape Thompson; **Identification:** identifiedBy: J. Salmela; **Event:** eventDate: 1961-7-29; **Record Level:** institutionCode: CNC
- **Type status:** Other material. **Occurrence:** recordedBy: R.E. Leech; individualCount: 1; sex: male; **Location:** country: USA; verbatimLocality: Alaska, Denali Highway, Mi. 31; minimumElevationInMeters: 1370; **Identification:** identifiedBy: J. Salmela; **Event:** eventDate: 1962-7-22; **Record Level:** institutionCode: CNC
- **Type status:** Other material. **Occurrence:** recordedBy: P.J. Skitsko; individualCount: 1; sex: male; **Location:** country: USA; verbatimLocality: Alaska, Isabel Pass. Mi. 206, Richardson Highway; minimumElevationInMeters: 880; **Identification:** identifiedBy: J. Salmela; **Event:** eventDate: 1962-7-16; **Record Level:** institutionCode: CNC

#### Distribution

Holarctic, here reported for the first time from Canada. The species was described from the arctic coast of Russia (New Siberian Islands and around Taimur, Lundström 1915), and was later rediscovered from arctic Russia (Dickson, New Siberian Islands, Zaitzev 1994). The species is also reported from Poland (Pommern, Landrock 1940) and Germany (Bavaria, Plassmann and Schacht 1999) and from Alaska in the USA (Hackman et al. 1988). However, the record from Germany is erroneous (misidentification, the male specimen is an undescribed species close to *B. nigricoxa* Staeger and *B. cincticornis* (Walker), det. J. Salmela) and the Polish record should also be re-examined and verified. Furthermore, the record of *B. birulai* larvae from Port Barrow, Alaska originally implicated in causing human myiasis to an entomologist (Casterline 1954), should be treated with caution. Larva of *B. birulai* has not been described and it remains unclear whether *B. birulai* or some other species was involved.

### Boletina pallidula

Edwards, 1925

Boletina pallidula Edwards, 1925: 573 (Edwards 1925)
Boletina subnitidula Sasakawa, 1994: 298 (Sasakawa 1994), syn. n.

#### Taxon discussion

*Boletina pallidula* was described by Edwards (Edwards 1925) from the British Isles, and the male hypopygium has been later illustrated by Hutson et al. 1980 and Zaitzev 1994. The species has a European range, including Fennoscandia, northern Russia, Central Europe, Bulgaria and Hungary (Chandler 2014). Sasakawa (Sasakawa 1994) described *B. subnitidula* from the Chiba prefekture in Japan based on material collected from *Arisema serratum* flowers. Sasakawa's description is very detailed, including illustrations on the male hypopygium, and hence we propose it as a junior synonym of *B. pallidula*. In his discussion on *B. subnitidula* Sasakawa (Sasakawa 1994, p. 300) does not mention *B. pallidula*; instead, he compares his new species to rather distant species *B. nitida* Grzegorzek and *B. dispecta* Dziedzicki. It is likely that Sasakawa was not aware of *B. pallidula* while describing *B. subnitidula*.

## Supplementary Material

Supplementary material 1 List of described Boletina species Data type: data sheet
Brief description: A list of described *Boletina* species, including biogeographic range and preliminary comments on taxonomy.
File: oo_71120.xls Jukka Salmela

Supplementary material 2 Ecology of Boletina species, larval records or rearings Data type: data sheet
Brief description: Ecological information on *Boletina* immature stages (larval records or rearings), extracted from literature.
File: oo_71121.xls Jukka Salmela

XML Treatment for Boletina valteri

XML Treatment for Boletina kullervoi

XML Treatment for Boletina hyperborea

XML Treatment for Boletina nuortti

XML Treatment for Boletina arctica

XML Treatment for Boletina borealis

XML Treatment for Boletina birulai

XML Treatment for Boletina pallidula

## Figures and Tables

**Figure 2a.**
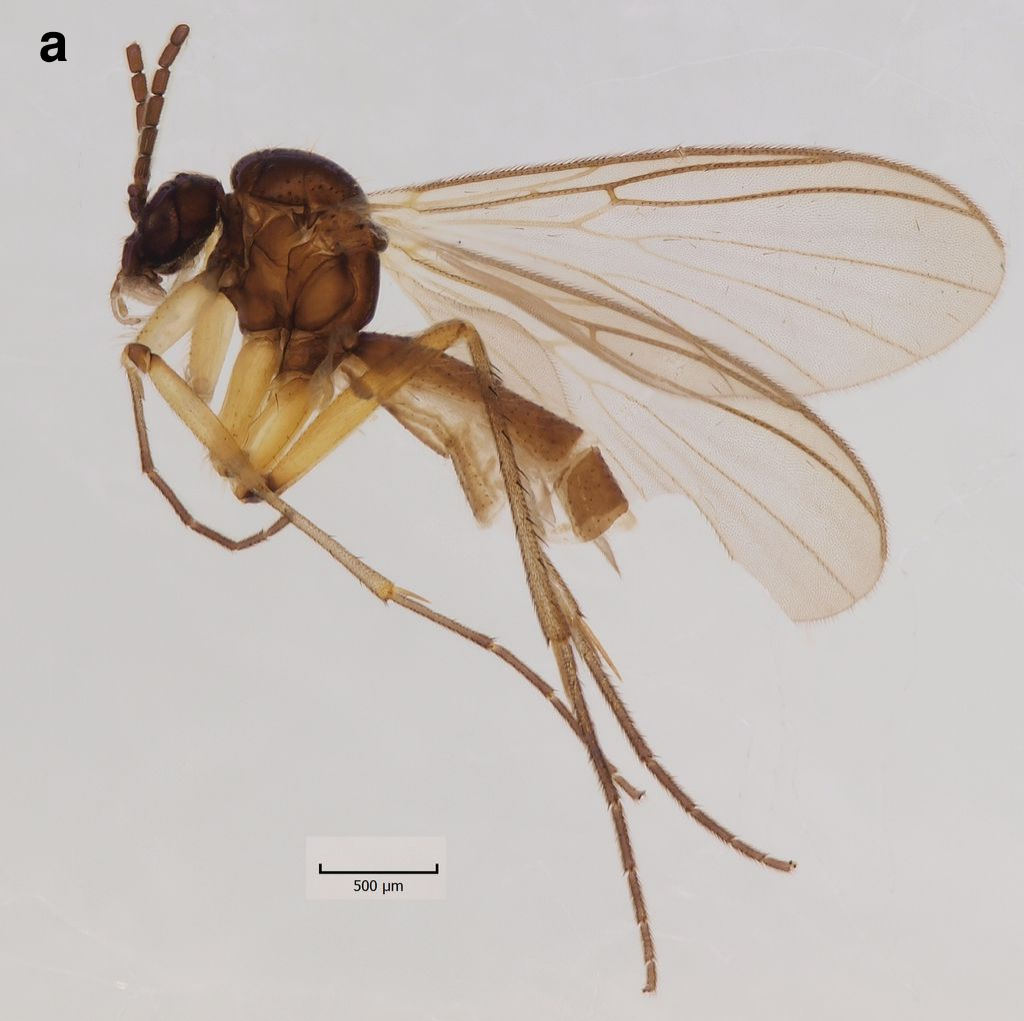
Habitus, lateral view.

**Figure 2b.**
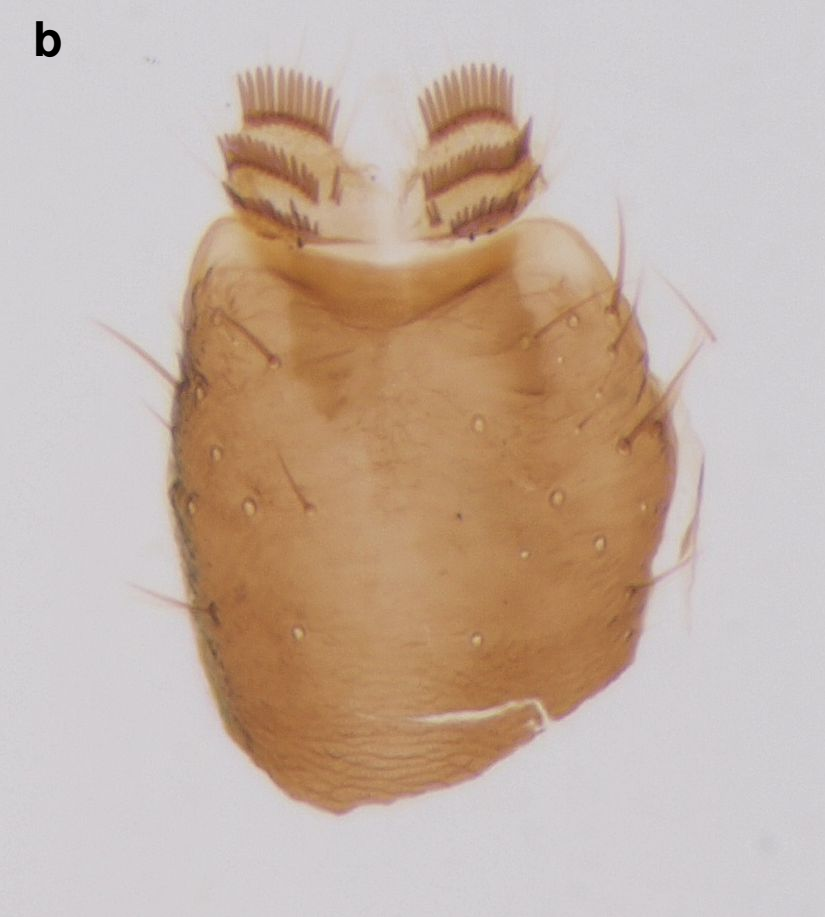
9th tergite and cerci, dorsal view.

**Figure 3a.**
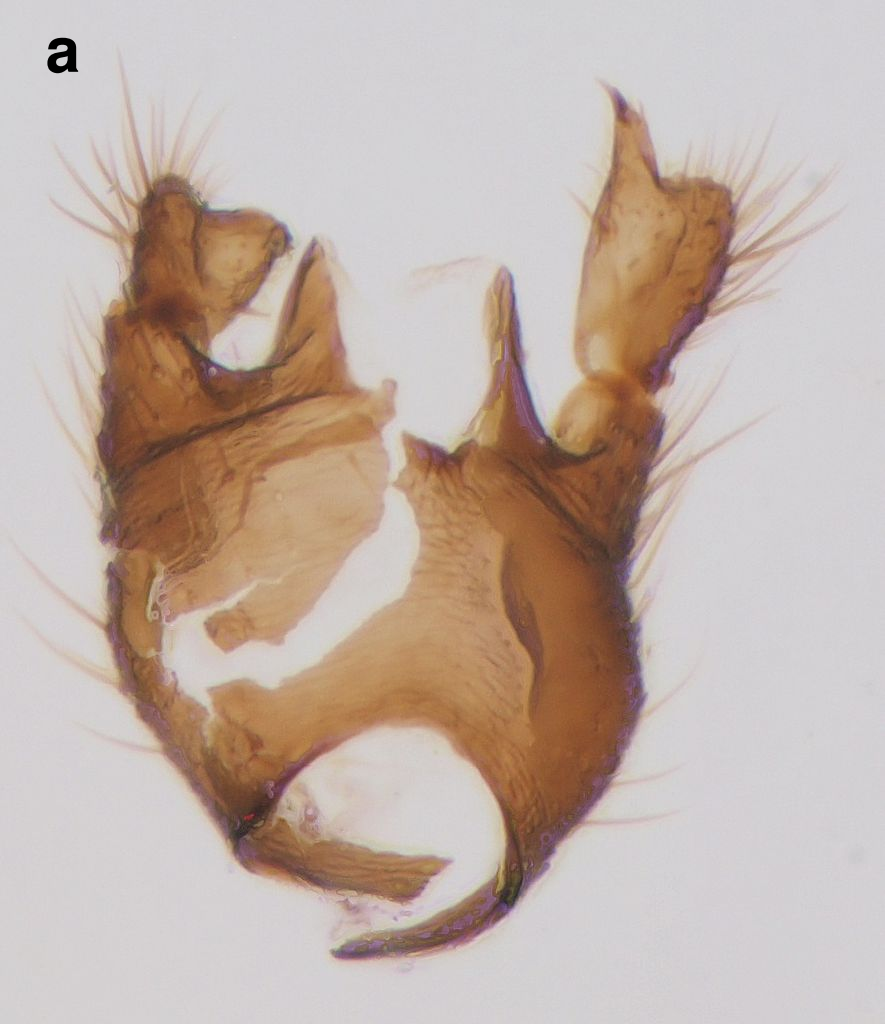
Hypopygium, dorsal view.

**Figure 3b.**
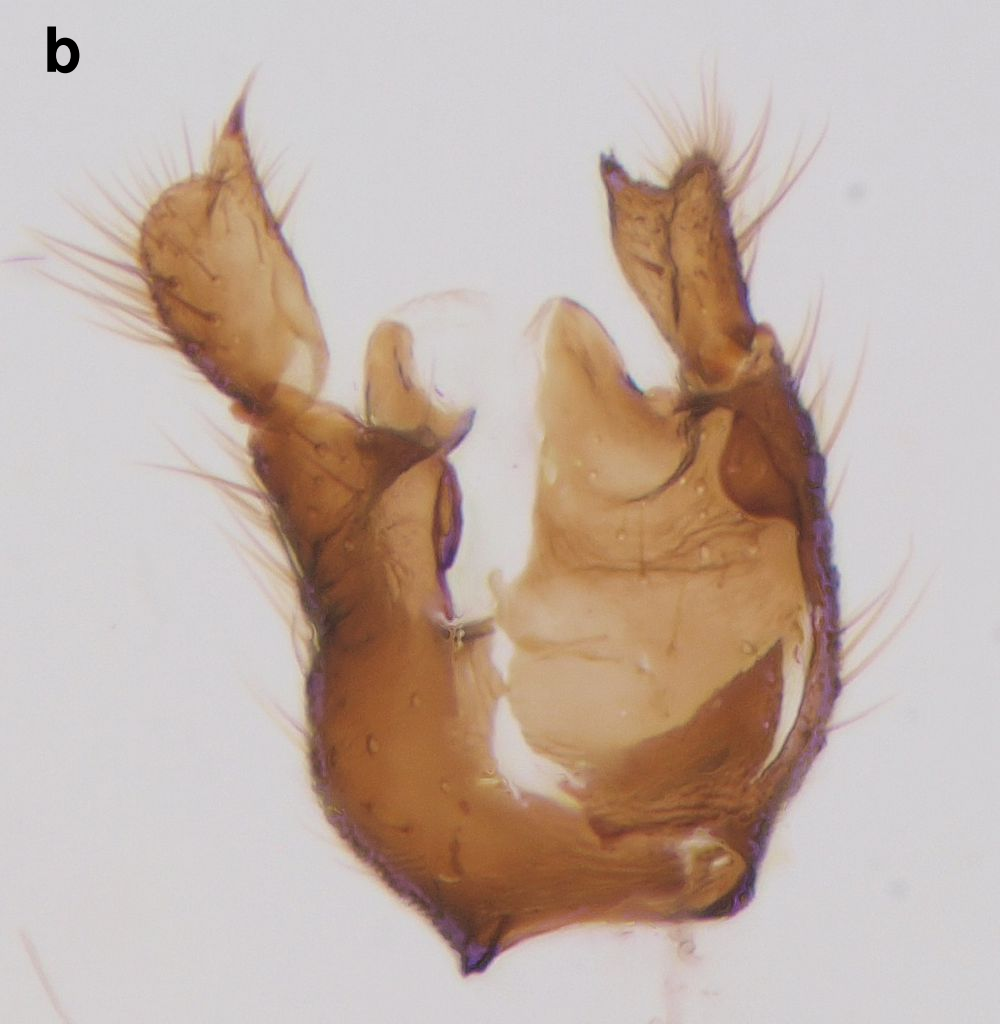
Hypopygium, ventral view.

**Figure 3c.**
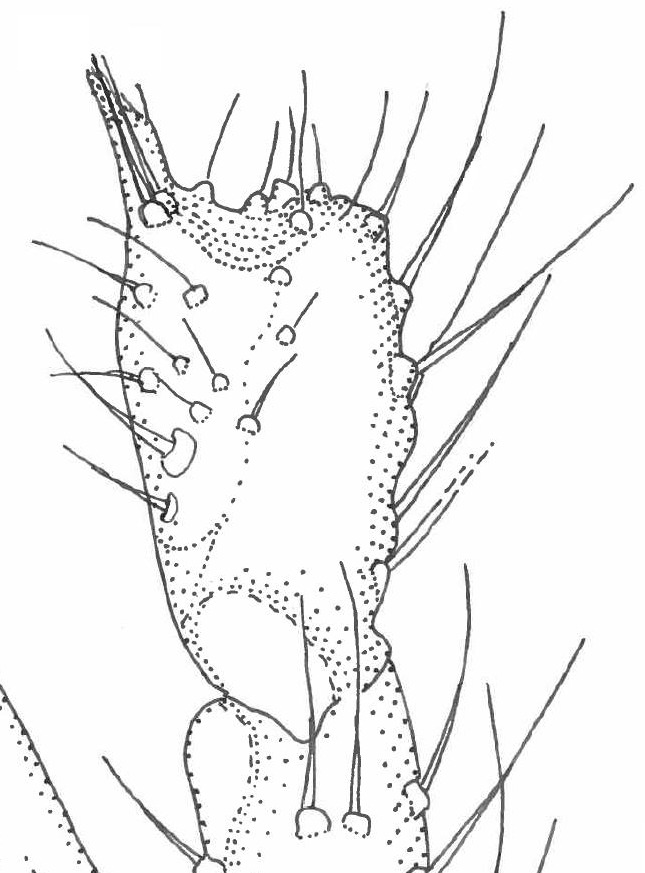
Gonostylus, dorsal view.

**Figure 3d.**
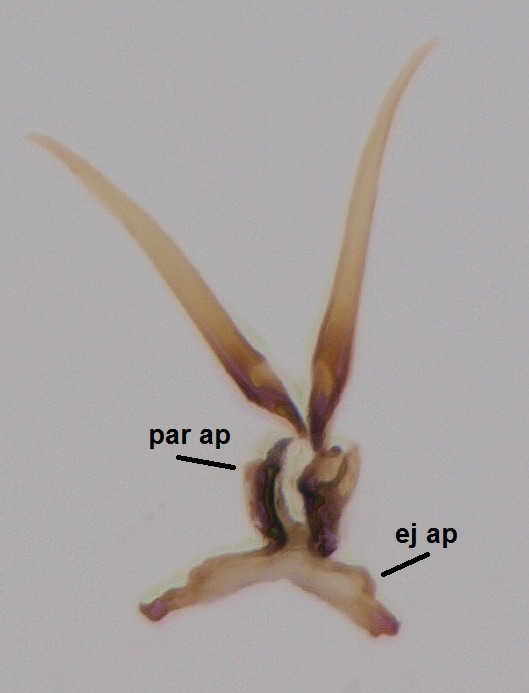
Aedeagal complex, dorsal view.

**Figure 3e.**
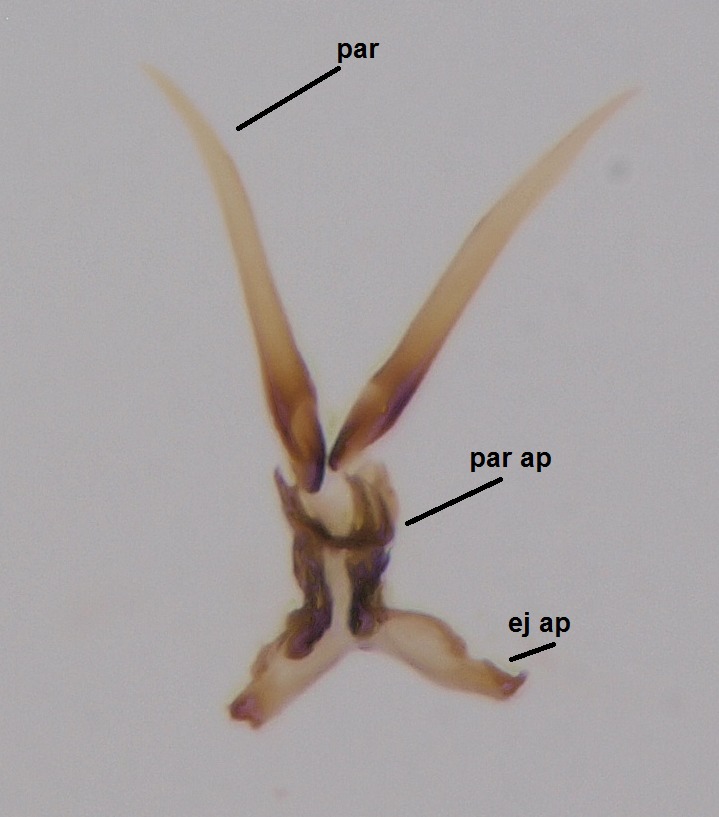
Aedeagal complex, ventral view.

**Figure 3f.**
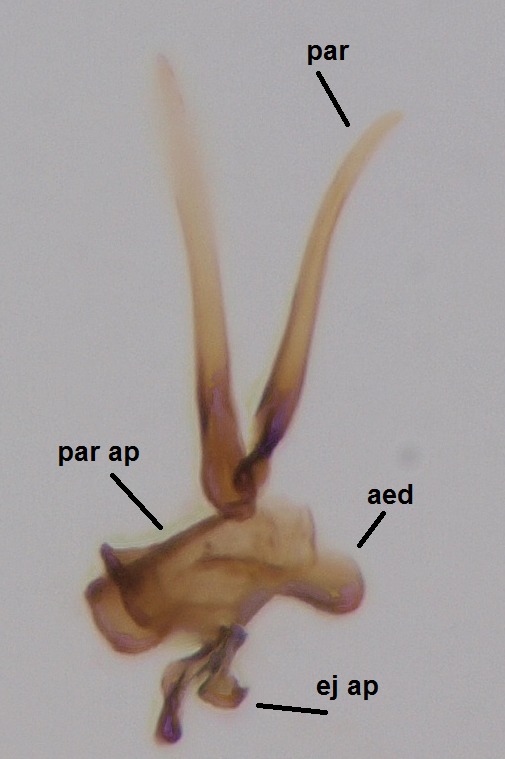
Aedeagal complex, lateral view.

**Figure 5a.**
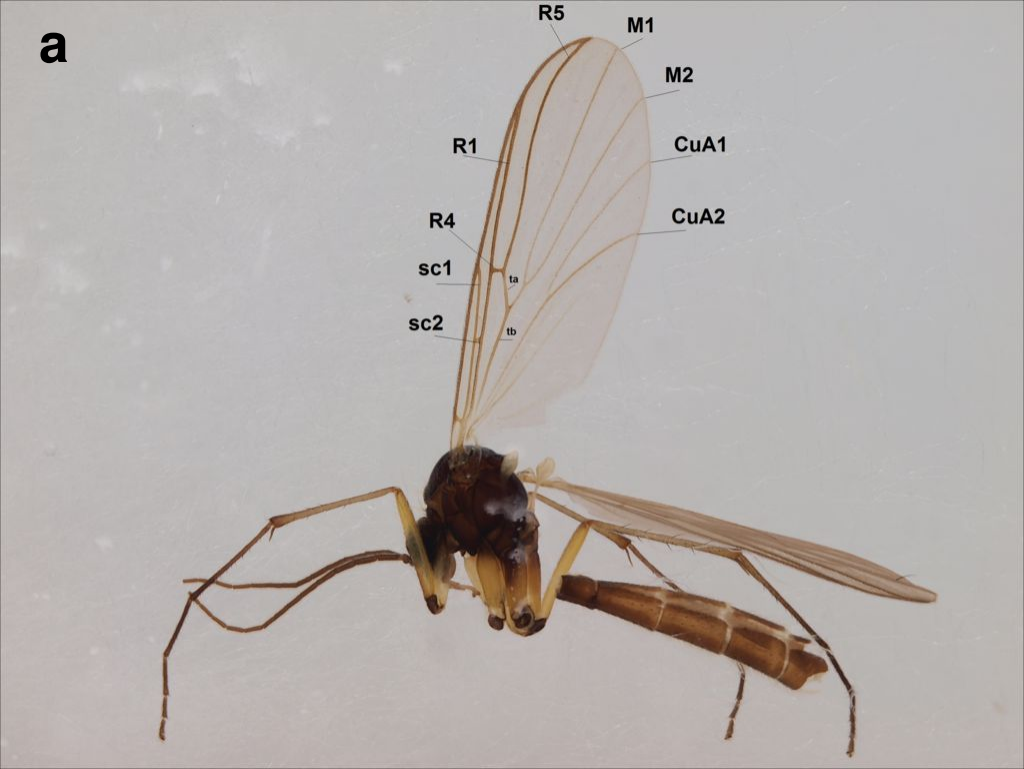
Lateral view.

**Figure 5b.**
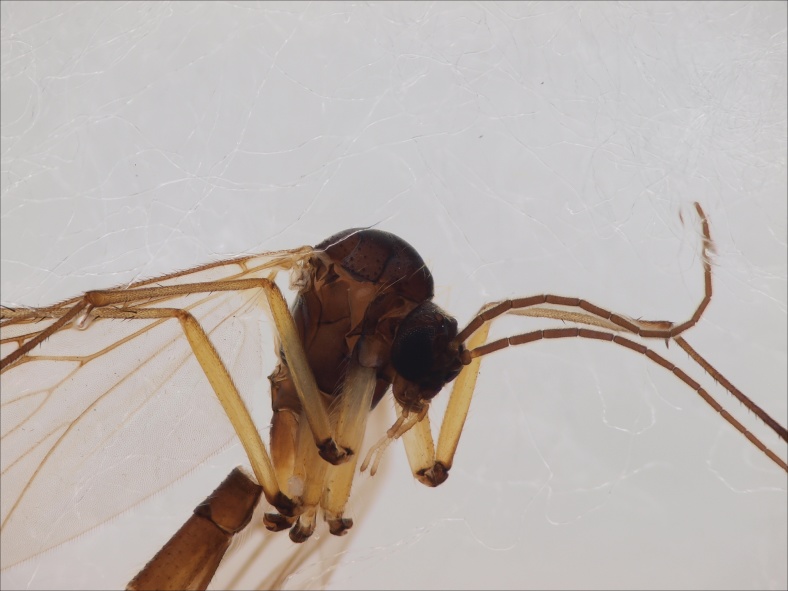
Thorax and head, antero-lateral view.

**Figure 6a.**
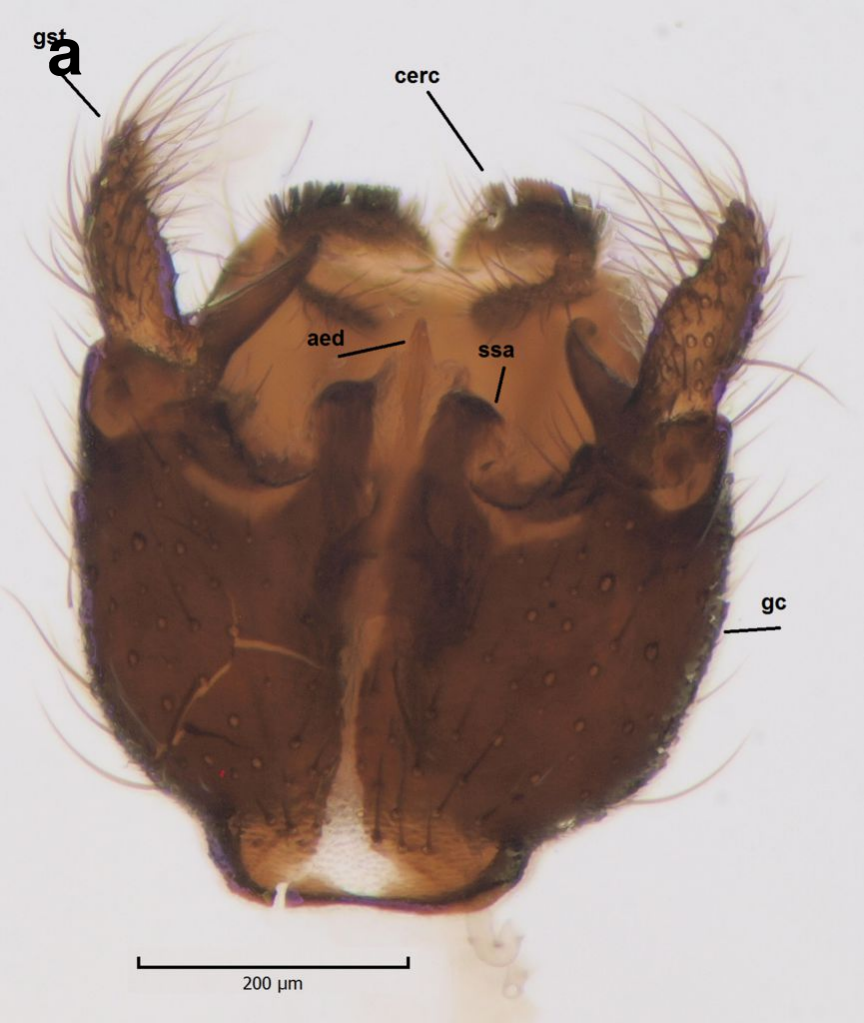
Hypopygium, ventral view.

**Figure 6b.**
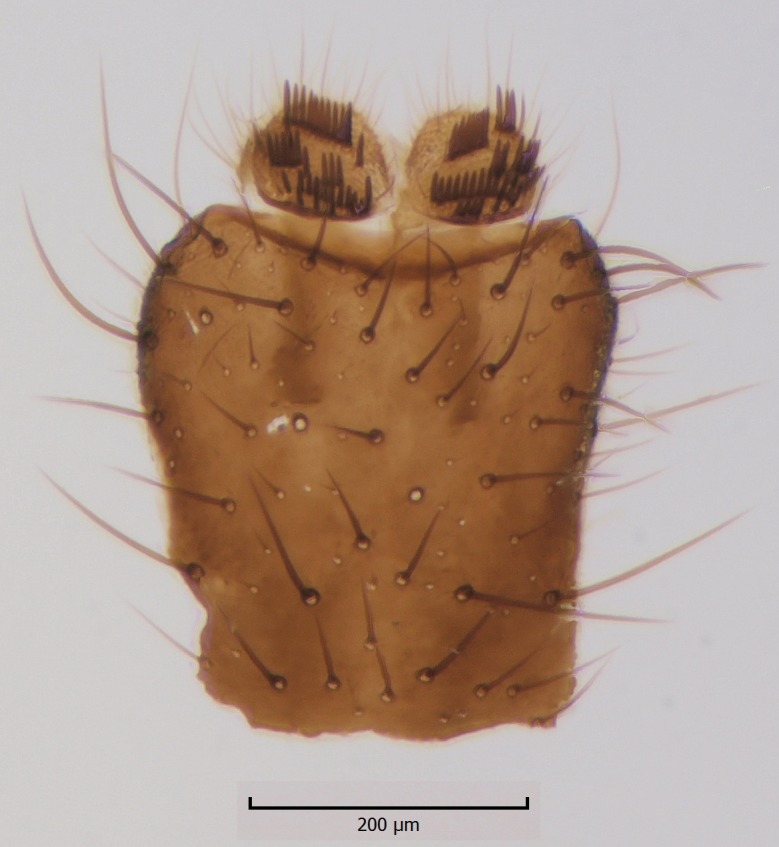
9th tergite and cerci, dorsal view.

**Figure 6c.**
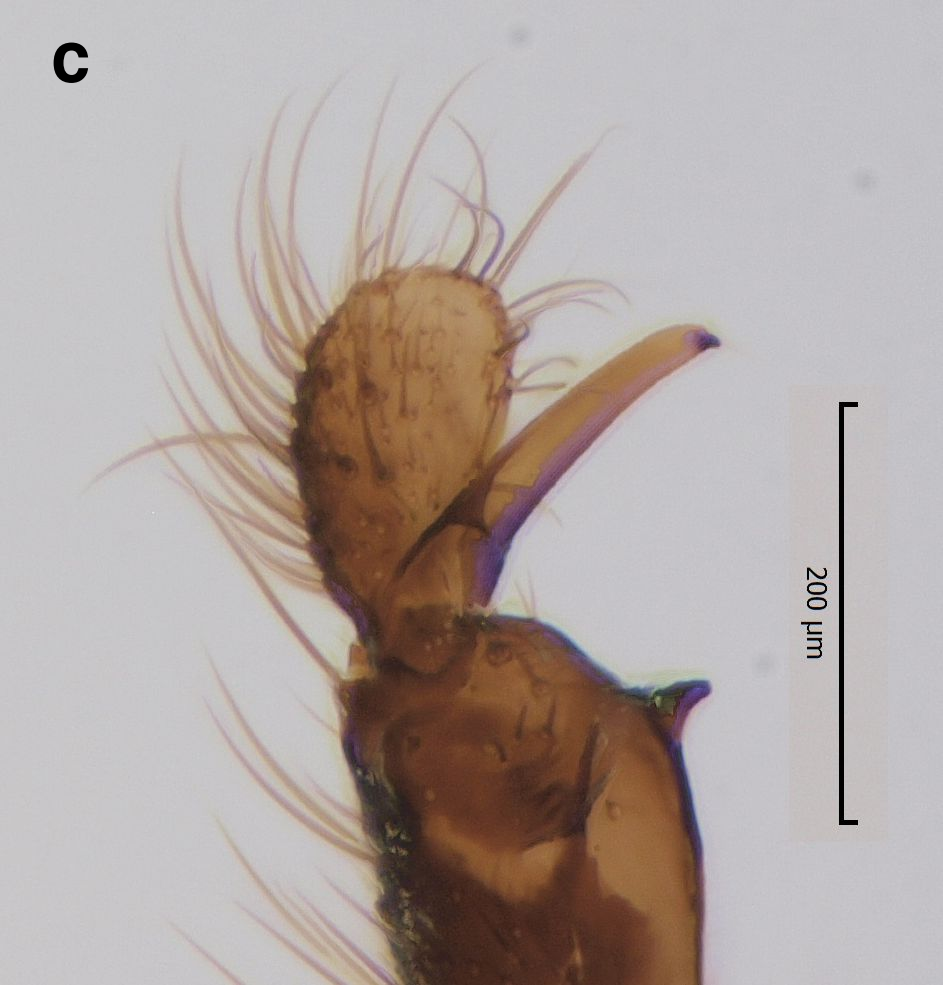
Gonostylus, inner view.

**Figure 6d.**
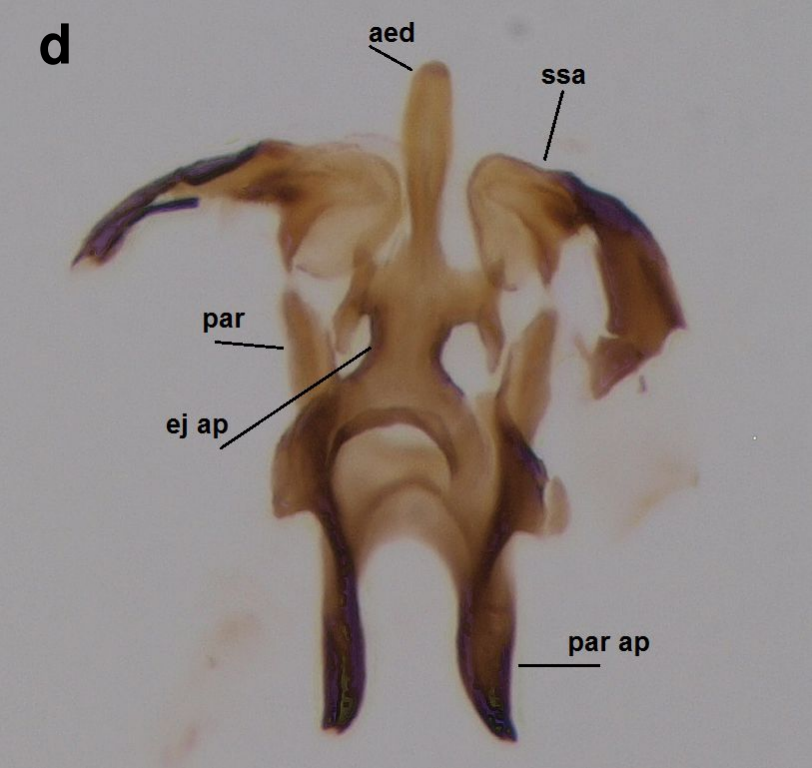
Aedeagal complex, dorsal view.

**Figure 6e.**
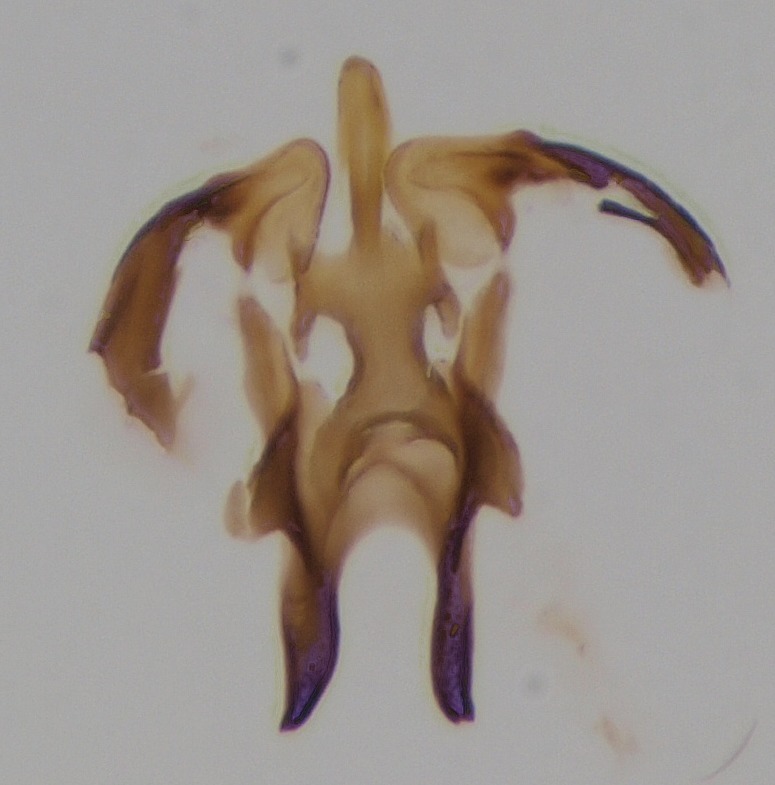
Aedeagal complex, ventral view.

**Figure 6f.**
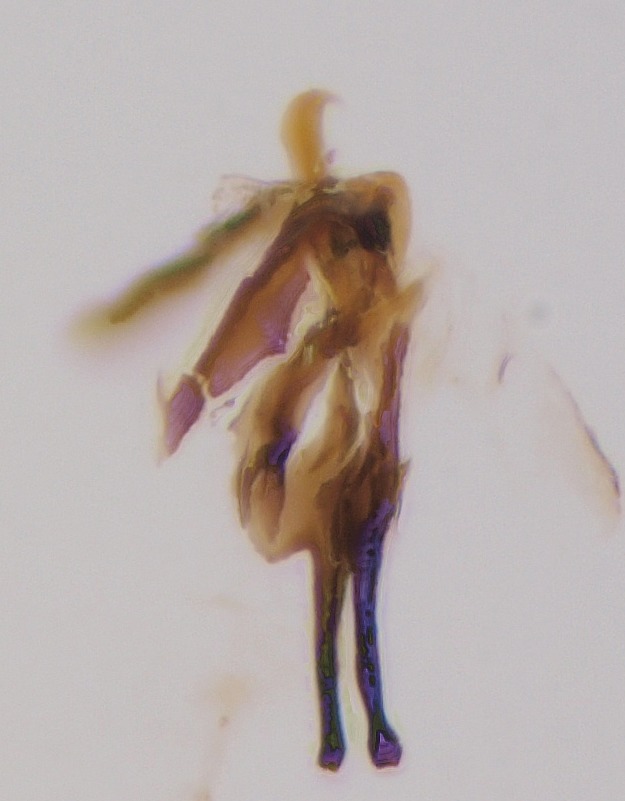
Aedeagal complex, lateral view.

**Figure 8a.**
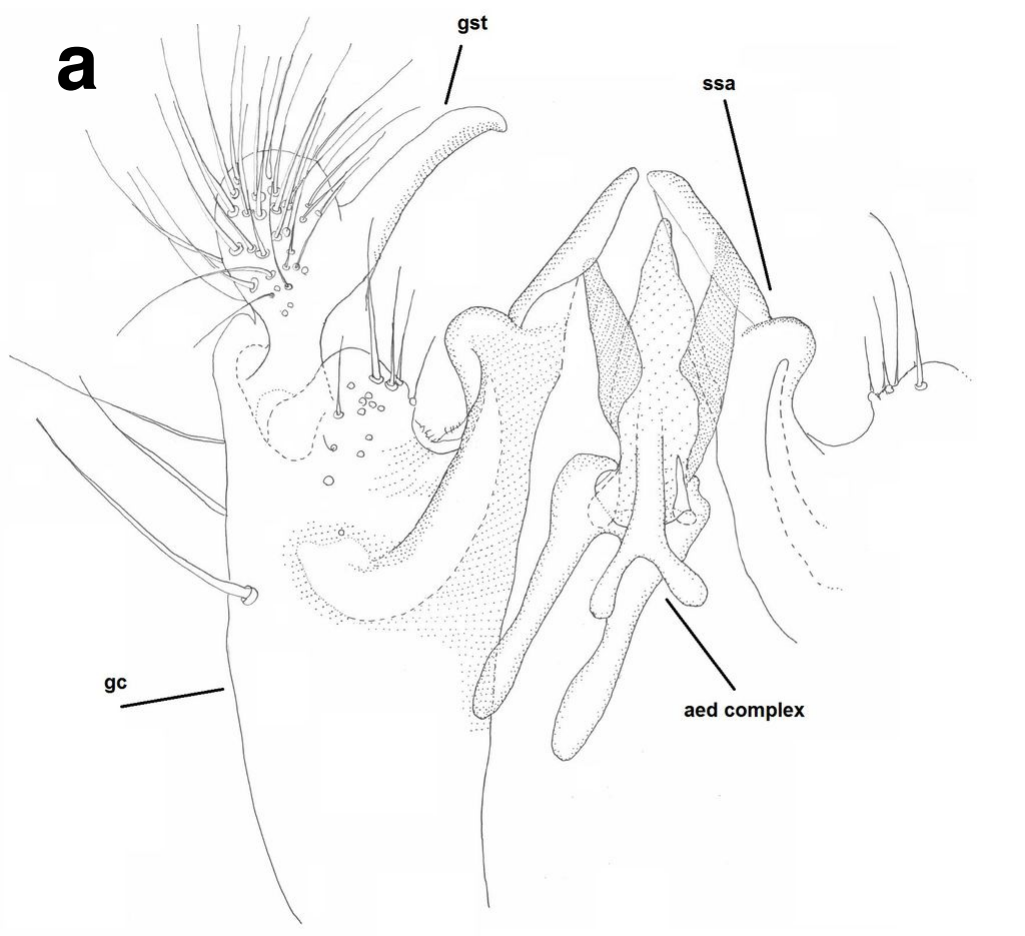
Hypopygium, ventral view.

**Figure 8b.**
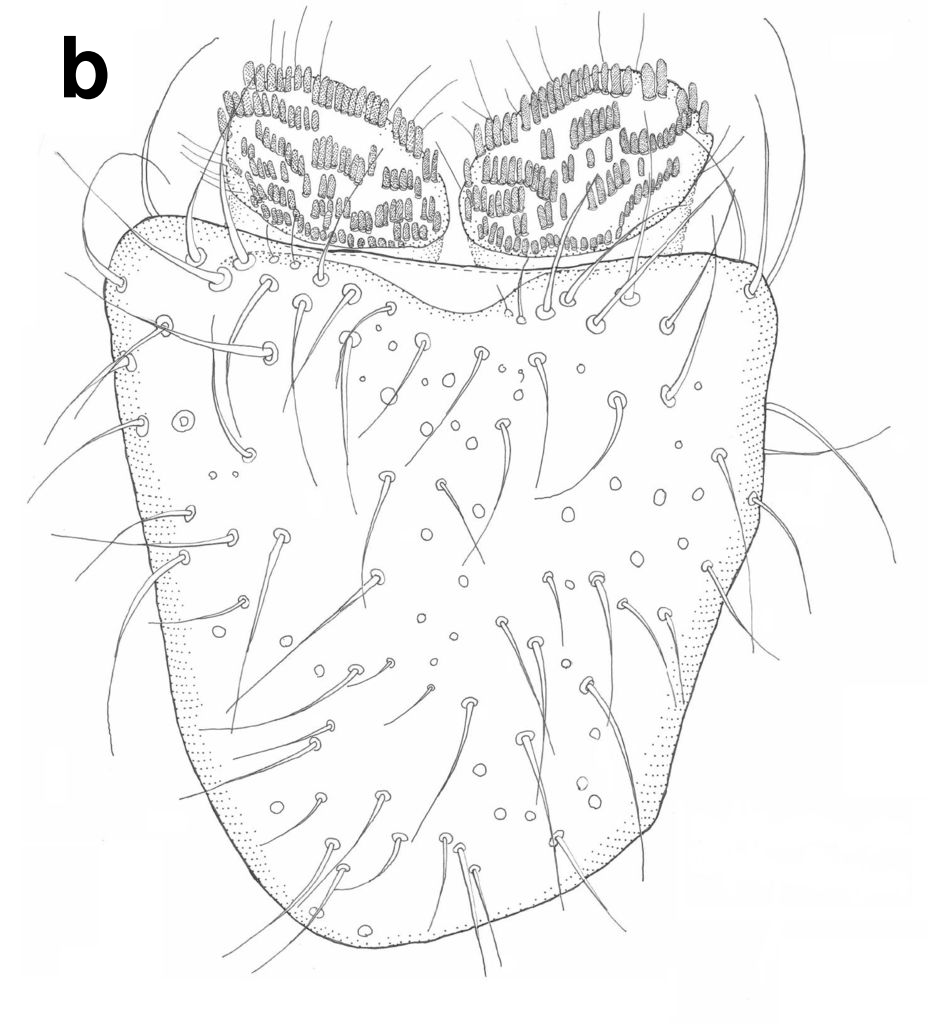
9th tergite and cerci, dorsal view.

**Figure 8c.**
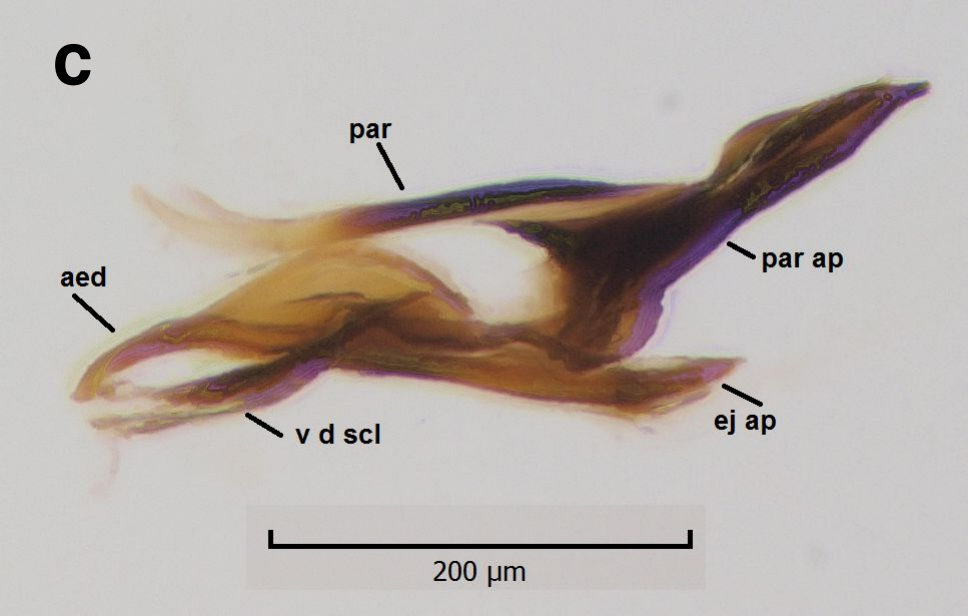
Aedeagal complex, lateral view.

**Figure 8d.**
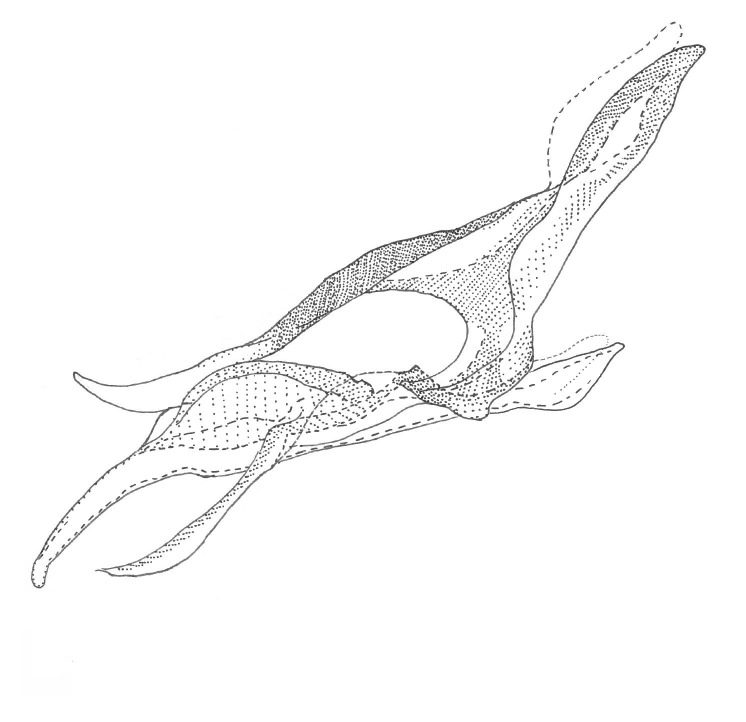
Aedeagal complex, lateral view.

**Figure 8e.**
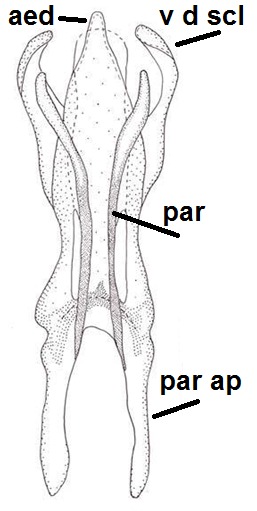
Aedeagal complex, dorsal view.

**Figure 8f.**
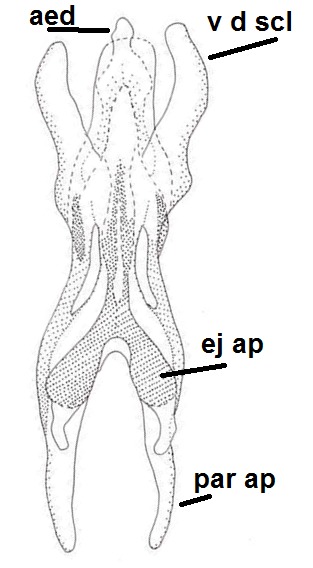
Aedeagal complex, ventral view.

**Figure 9a.**
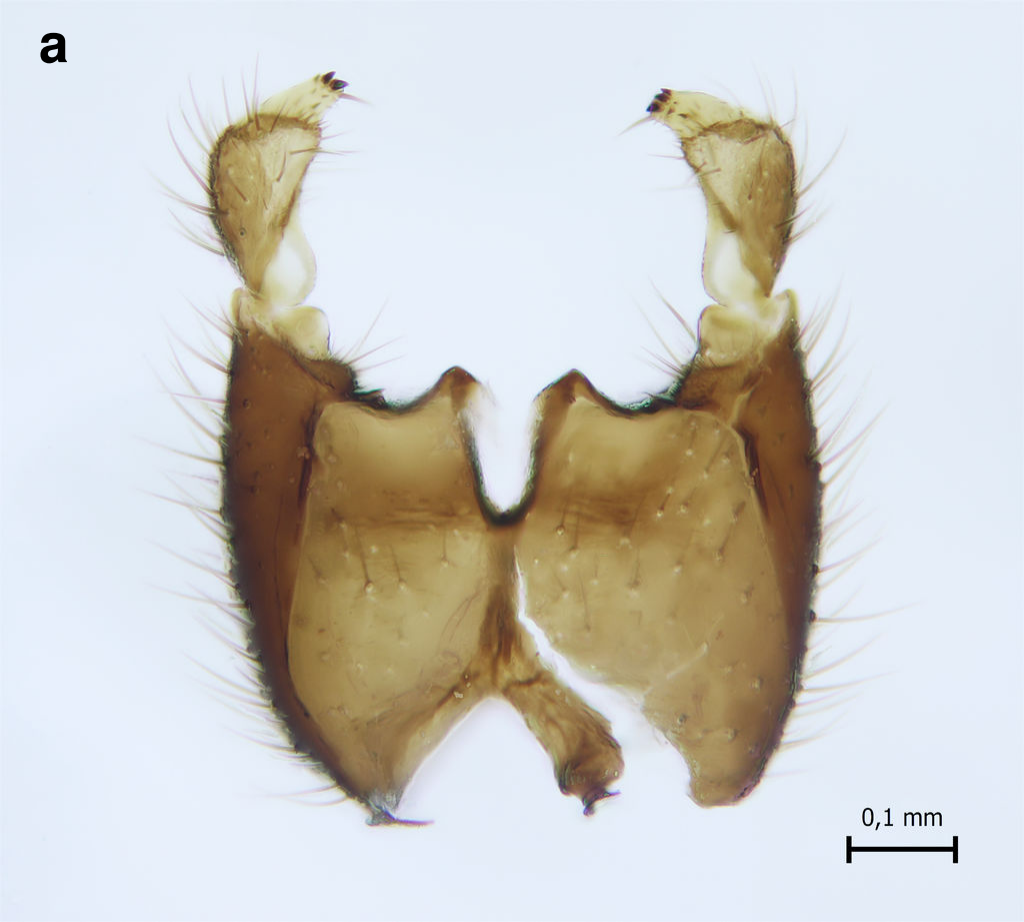
Hypopygium, ventral view.

**Figure 9b.**
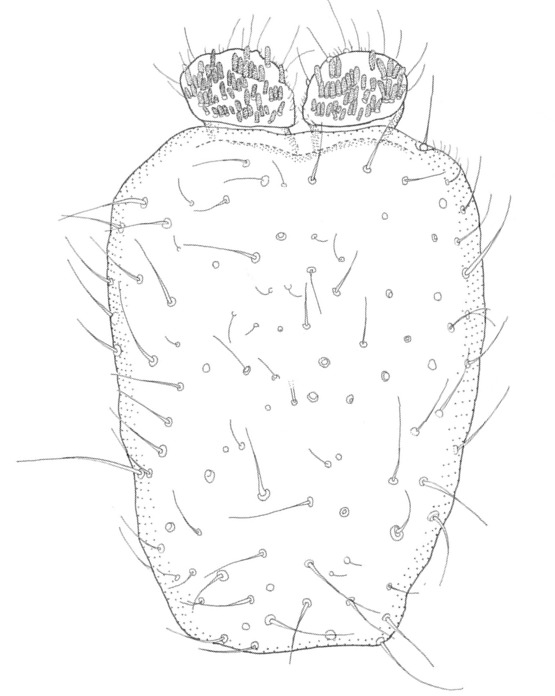
9th tergite, dorsal view.

**Figure 10a.**
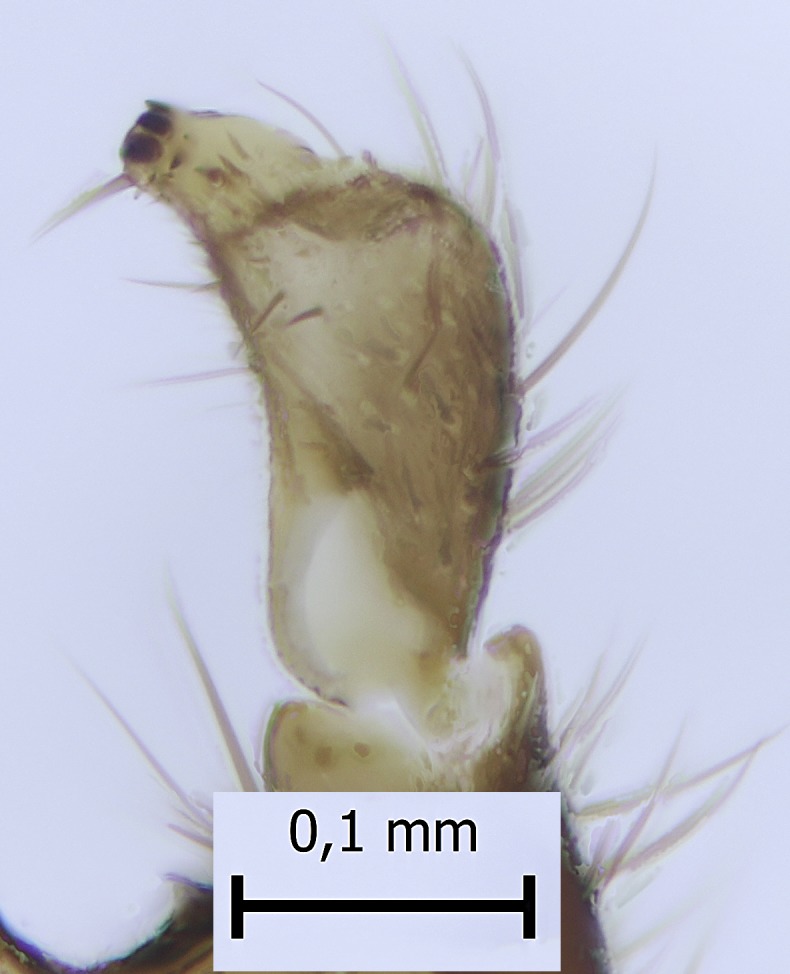
Gonostylus, ventral view.

**Figure 10b.**
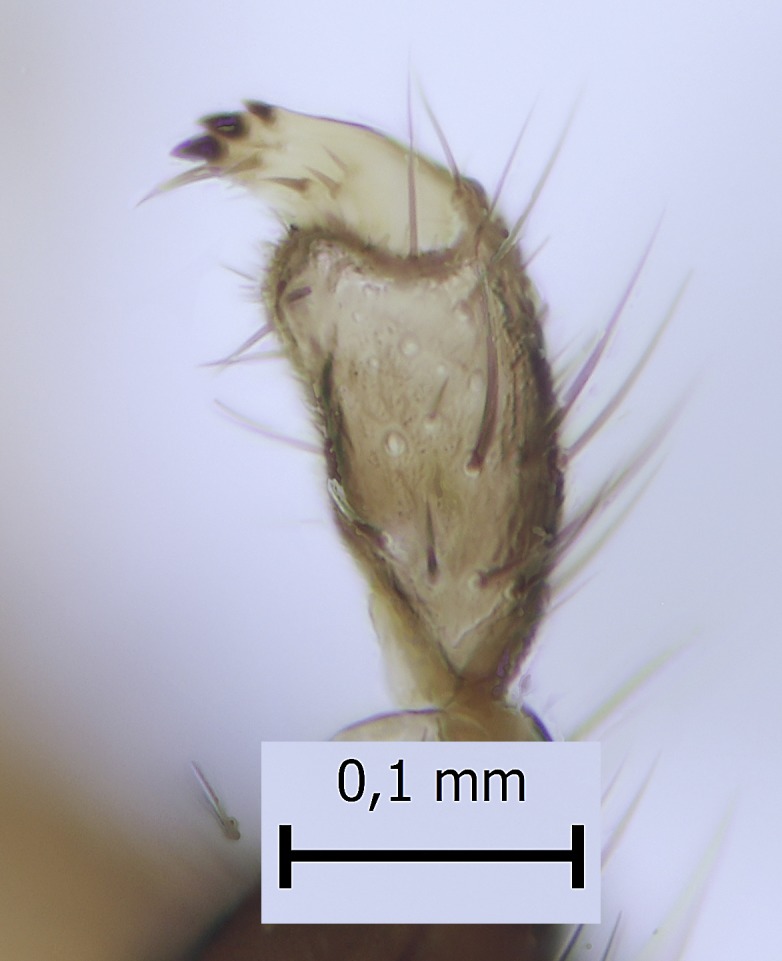
Gonostylus, dorsal view.

**Figure 10c.**
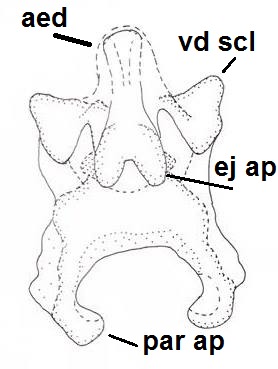
Aedeagal complex, ventral view.

**Figure 10d.**
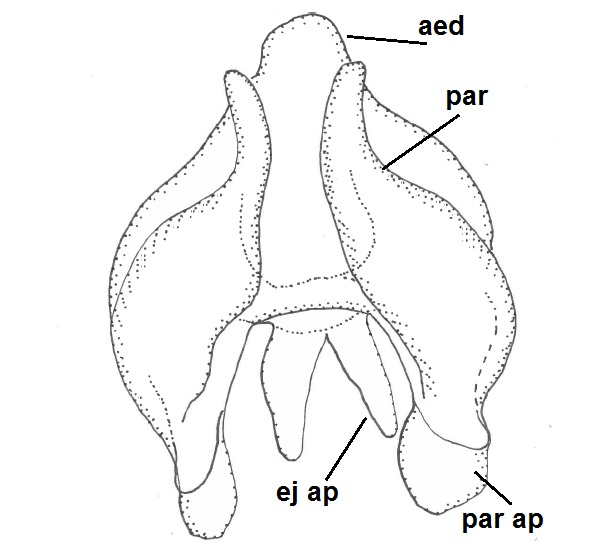
Aedeagal complex, dorsal view.

**Figure 10e.**
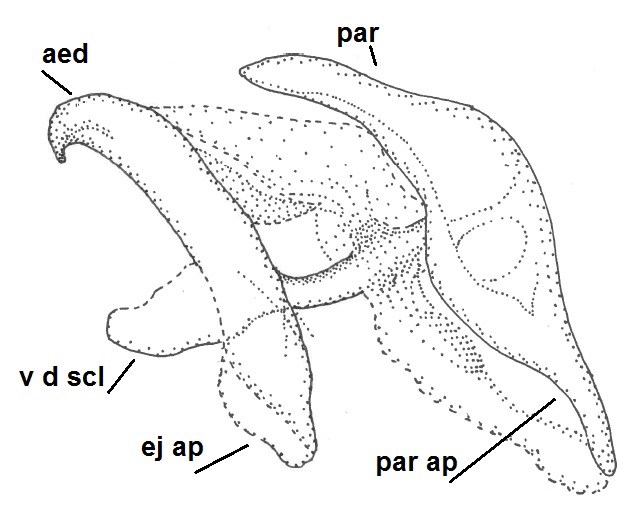
Aedeagal complex, lateral view.

**Figure 11a.**
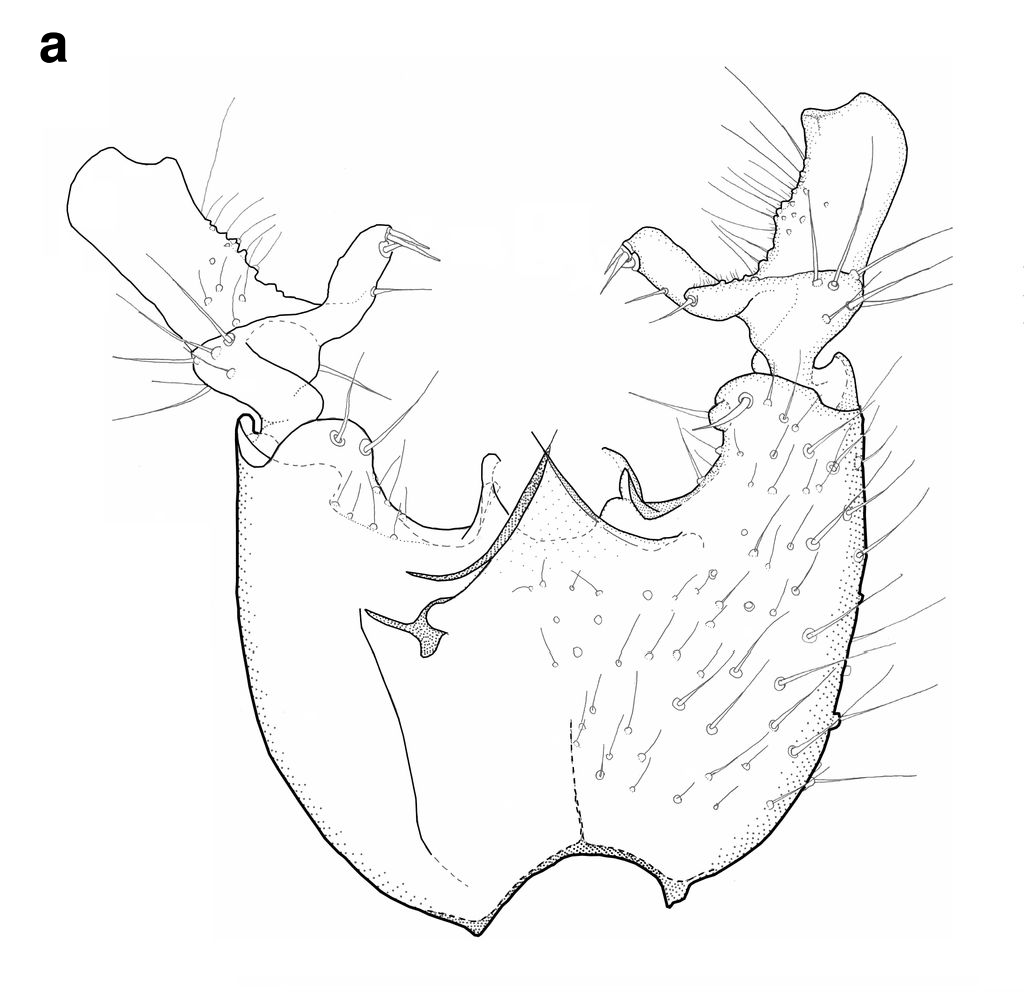
Hypopygium, ventral view. Sternal submedian appendages of gonogoxites are omitted.

**Figure 11b.**
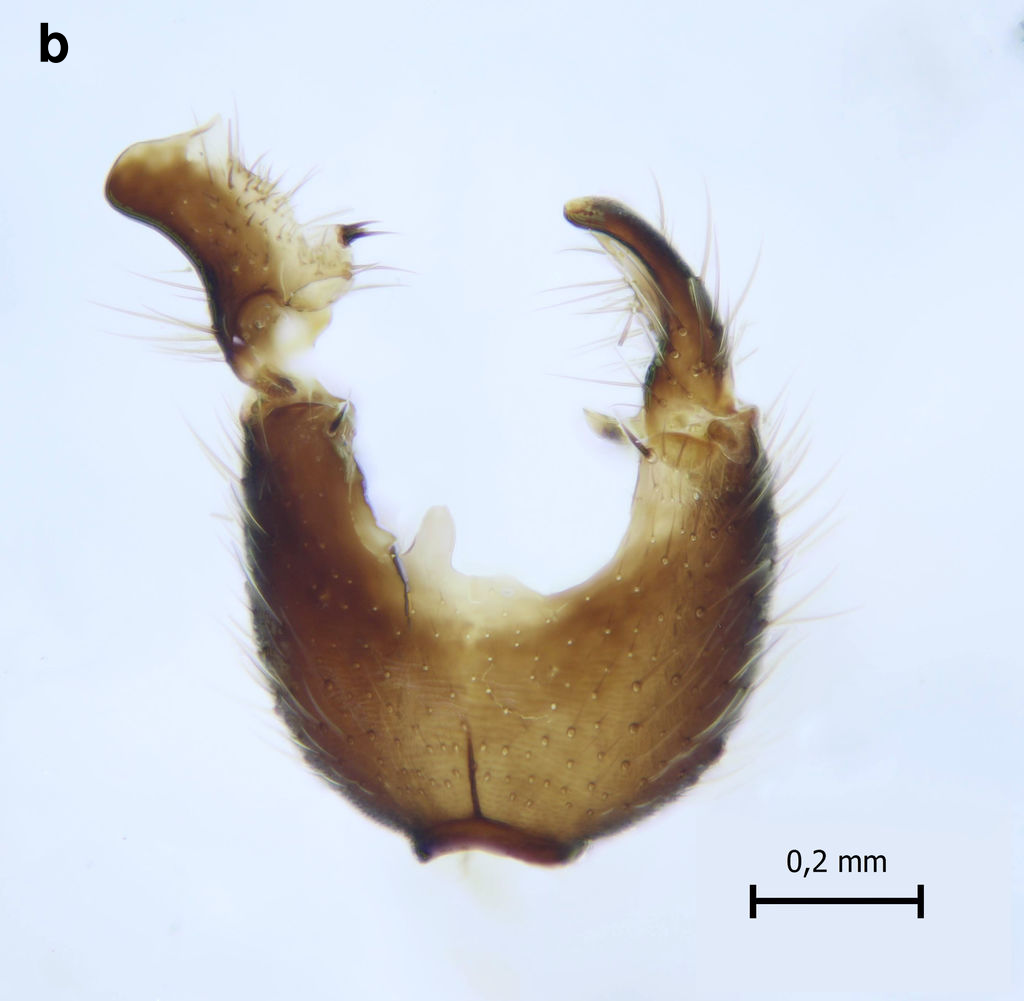
Hypopygium, ventral view. Sternal submedian appendages of gonogoxites are omitted.

**Figure 11c.**
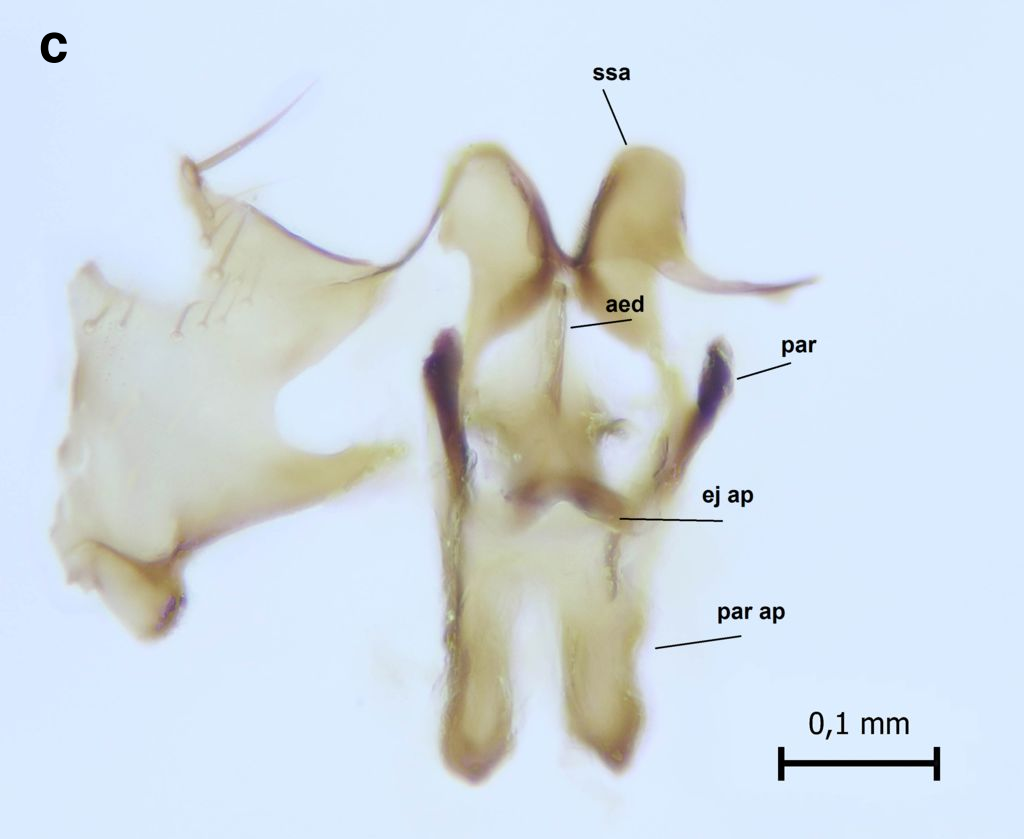
Aedeagal complex, dorsal view.

**Figure 11d.**
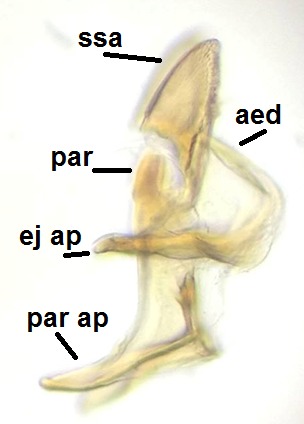
Aedeagal complex, lateral view.

**Table 1.** Measurements (µm) of the four new *Boletina* species.

**trait**	**B. hyperborea**	**B. kullervoi** (n=2)	**B. nuortti**	**B. valteri**
scape	120	120-125	80	80
pedicel	90	70-75	60	60
1st flagellomere	310	270-290	160	120
2nd flagellomere	260	220	170	120
width of 2 flagellomere	60	60	50	60
fore tibia	1325	1325	850	710
fore 1st tarsomere	900	1150	725	470
mid tibia	1725	1875	1125	n.a.
mid 1st tarsomere	1025	1375	750	n.a.
hind tibia	2530	2375	1500	1425
hind 1st tarsomere	1150	1325	925	725
ta	480	380-400	200	130
M-stem	450	250-310	400	230
